# Tuning High-Density Polyethylene Microstructure and Properties from Known Distributions of Dynamic Bonds

**DOI:** 10.1021/jacs.5c13586

**Published:** 2025-12-04

**Authors:** Christopher B. Cooper, McKenzie L. Coughlin, Polette J. Centellas, Aaron A. Burkey, Kalman B. Migler, Jonathan E. Seppala, Edwin P. Chan, Chad R. Snyder, Sara V. Orski

**Affiliations:** Materials Science and Engineering Division, 10833 National Institute of Standards and Technology, Gaithersburg, Maryland 20899, United States

## Abstract

Over 50% of plastic waste comes from a single class of polymers called polyolefins. Most recycling strategies fail to preserve the broad molecular mass distribution of these polyolefins, from which they derive their unique combination of processability and mechanical strength. Here, we show that incorporation of urethane-based dynamic bonds into high-density polyethylene (HDPE) can circumvent this issue, by strengthening the amorphous phase through interchain supramolecular interactions, as opposed to traditional entanglements from high molecular mass chains. We show that many key properties of these dynamic HDPE polymers, including percent crystallinity, melting temperature, lamellae and amorphous thicknesses, and bond association, are determined by the distribution of bond-to-bond spacings along the chain backbone and are predicted using polymer physics theories. Moreover, we find that a mixed backbone HDPE dynamic polymer exhibits mechanical properties that exceed HDPE and approach the strain-hardening behavior seen in ultrahigh molecular weight polyethylene along with a unique display of long-range supramolecular order that persists in the melt state. This work illuminates key principles governing how the placement of dynamic bonds influences bulk material properties and provides a framework for toughening semicrystalline polymers and designing chemical recycling processes based on controlling bond-spacing distributions.

## Graphical abstract

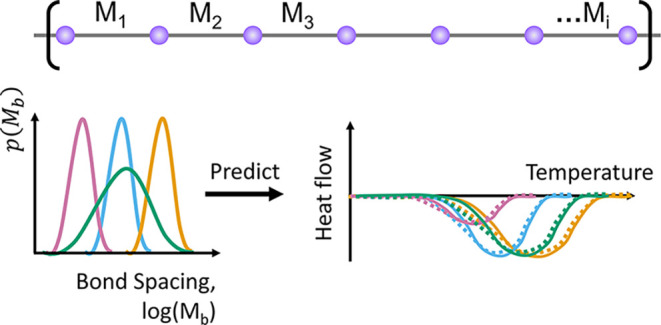

## Introduction

The use of polyolefin thermoplastics within the US, including high-density polyethylene (HDPE), low-density polyethylene (LDPE), linear low-density polyethylene (LLDPE), and polypropylene (PP) resulted in about 15,000 U.S. tons (or ∼30 million pounds) *collected* for recycling in 2018 and 22,000 U.S. tons (or ∼43.2 million pounds) discarded.^1^ This large amount of discarded plastic, including losses between collection and recycling, has a significant impact in community waste management, economic opportunity losses, and the environment.^2–5^ Even as efforts to improve collection, sortation, and mechanical recycling of polyolefins are emphasized, multiple technological and engineering approaches are needed to address the large scale of polyolefin waste, which remain highly desirable materials due to their chemical resistance and excellent mechanical and barrier properties.

Chemical recycling can address many of the limitations of direct mechanical recycling, particularly heterogeneous mixtures that may result in phase separation and inferior mechanical properties, previously recycled resins, undesired cross-linking and chain scission during reprocessing, and separation and purification challenges. Different approaches have been investigated to chemically recycle plastic waste including: conversion back to monomers and repolymerization,^6–9^ additives into virgin plastics to introduce cleavable groups e.g., esters, alkenes, or thionolactones into the backbone,^10–12^ deconstruction to oils and other products,^13–15^ and upcycling by direct functionalization or oligomerization and recombination into mechanically recyclable products.^16–22^ The latter approach, in which polyolefin waste is upcycled into a new product that can then be mechanically recycled, is promising since it works for both existing and future waste and also reduces the total energy use and carbon footprint of the process.^23,24^

Nonetheless, this upcycling approach is constrained by a significant limitation–chain scission during reprocessing leads to selective loss of high molar mass chains, producing inferior mechanical properties after recycling.^25^ Moreover, any mechanism that reduces polyolefin chains into shorter oligomers, through processes such as dehydrogenation and cross metathesis,^17,26^ and then repolymerizes them with degradable units will remove the highest molecular mass chains (Figure 1a). Thus, an open question is how to design an upcycling process (or more broadly, a toughening mechanism) that does not require a fraction of high molecular mass chains in the final polymer to achieve competitive mechanical properties.

**Figure 1:**
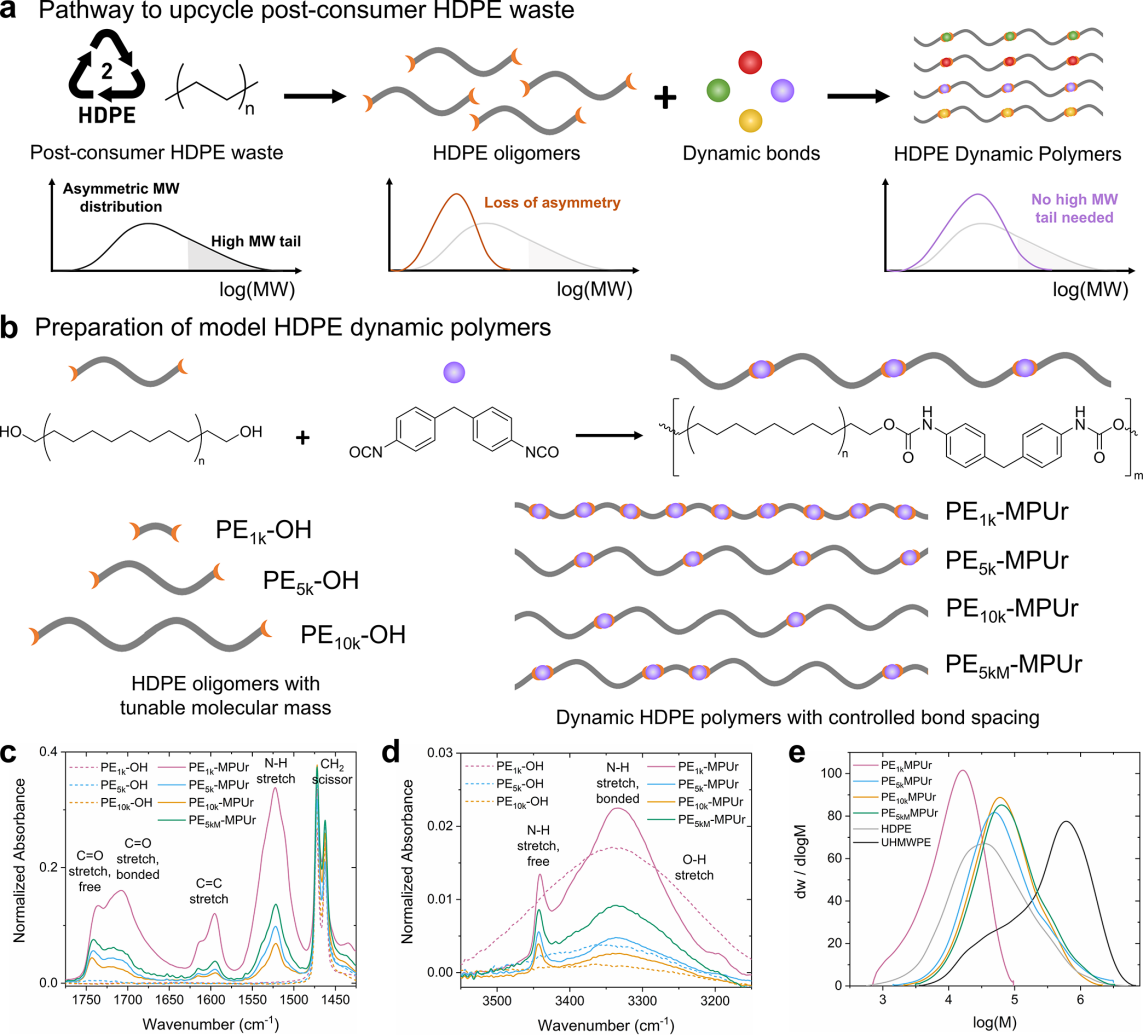
Design and synthesis of dynamic HDPE polymers. (a) Schematic pathway showing loss of high molar mass chains during breakdown and repolymerization with dynamic bonds (or other functional groups). (b) Synthesis of model dynamic HDPE polymers with controlled bond placement. (c, d) FTIR of the dihydroxy-terminated HDPE (dashed lines; PE_1k_OH, pink; PE_5k_OH, blue; PE_10k_OH, gold) before polymerization and of the dynamic HDPE polymers (solid lines; PE_1k_MPUr, pink; PE_5k_MPUr, blue; PE_10k_MPUr, gold; PE_5kM_MPUr, green) after polymerization. (e) Differential molar mass distribution for PE_1k_MPUr (pink), PE_5k_MPUr (blue), PE_10k_MPUr (gold), PE_5kM_MPUr (green), HDPE (light gray), and UHMWPE (dark gray) derived from molar mass units of g/mol.

Moreover, the increasing modification of polyolefin materials with different chemistries has raised questions about the structure–property relationships arising from functional group addition. Such relationships are needed to properly tune bulk properties and, ideally, to be able to predict them *a priori*. Quantifying tolerance for functional group integration and the distribution of functional groups on resultant polymer properties (mechanical and in the melt) can help streamline research focus areas and facilitate shorter paths to commercial adaptation. This will allow these new methods to be strategically targeted to fill in gaps in expanding an efficient materials economy, where integrated approaches can guide creative, functional chemistries into upcycling polyolefins into high value applications, integrate with industrial infrastructure, and facilitate rapid commercialization.

Here, we demonstrate a promising option based on the incorporation of dynamic bonds into HDPE (Figure 1a). We define a dynamic bond broadly to include any interchain interaction (noncovalent or covalent) with partner exchange at readily accessible temperatures.^27^ Incorporating such reversible interactions has enabled enhanced material properties including self-healing, high toughness, and shape memory behavior.^28,29^ In polyolefins, dynamic bonds can compatibilize immiscible waste plastics that show significant microphase separation, though they often dramatically reduce backbone crystallization.^30–32^ In this work, we show that promoting interchain interactions between dynamic bonds in the amorphous phase enhances the mechanical properties of moderate molar mass, functional polyolefins, analogous to the effect of increasing tie chains in highly entangled melts. Furthermore, through synthesizing a set of model HDPE dynamic polymers with different amounts and *sequences* of urethane-based dynamic bonds (Figure 1b), we show that defining a bond spacing distribution enables the prediction of key properties, including percent crystallinity, melting temperature, lamellae thickness, and degree of bond association, inspired by previous work demonstrating similar effects on functionalized HDPE with precisely controlled spacings.^33–35^ This bond spacing distribution is critical to the control of the polymer microstructure and mechanical properties, highlighting that a dynamic HDPE polymer with mixed backbone spacings can exhibit mechanical properties that readily exceed HDPE and approach the strain-hardening behavior seen in ultrahigh molecular weight polyethylene (UHMWPE), by combining the advantages of both short and long bond spacing lengths. Through X-ray scattering experiments, we show that this mixed backbone HDPE dynamic polymer displays long-range supramolecular order that persists even in the melt state. This work establishes structure–property relationships relating the molecular design of new functionalized polyolefins to their bulk thermal, mechanical, and structural properties, a key step forward to build the pathway to upcycle current and future plastic wastes.

## Results and Discussion

### Synthesis of Model Dynamic HDPE Polymers and Determination of Bond-Spacing Distributions

We selected high-density polyethylene (HDPE) as our model polyolefin backbone for dynamic bond incorporation and modified previously reported synthetic strategies to prepare telechelic hydroxy-functionalized HDPE macromonomers (PE–OH) with different molecular masses (see Experimental section).^36^ Briefly, we performed ring-opening metathesis polymerization on cyclooctene with controlled amounts of chain transfer agent to target specific molecular masses, and then performed hydrogenation and deprotection steps to convert the synthesized polycyclooctene (PCO) to PE–OH. We targeted three different number-average molecular masses (*M*_n_) of 1 kg/mol (named PE_1k_OH), 5 kg/mol (PE_5k_OH), and 10 kg/mol (PE_10k_OH) and confirmed their synthesis via proton nuclear magnetic resonance spectroscopy (^1^H NMR), Fourier transform infrared spectroscopy (FTIR), and size-exclusion chromatography (SEC) (Table 1 and Figures S1–S4 **PCO** and Figures S5–S9 **PE–OH**).

**Table 1:** Characterization of PE–OH Macromonomers, HDPE Dynamic Polymers, and HDPE Benchmarks

		HT-NMR	HT-SEC			DSC			
polymer	PE mass (%)	*M*_n_ (kg/mol)	*M*_n_ (kg/mol)	*M*_w_ (kg/mol)	*M*_z_ (kg/mol)	*T*_c_ (°C)	*T*_m_ (°C)	Δ*H*_m_ (J/g)	PE crystallinity by mass fraction (%)
PE_1k_–OH	96.9	1.5	0.96	1.7	2.8	113	123	261	92
PE_5k_–OH	99.4	5.4	4.3	8.6	14	119	131	278	95
PE_10k_–OH	99.7	13.5	9.5	22	38	121	133	261	89
PE_1k_-MPUr	80.0	-	6.9	17	29	89	107	63	27
PE_5k_-MPUr	95.2	-	34	150	720	112	123	150	54
PE_10k_-MPUr	97.6	-	39	120	350	115	128	171	60
PE_5kR_-MPUr	95.2	-	45	160	570	111	124	141	50
HDPE	100	-	18	110	550	119	135	221	75
UHMWPE	100	-	103	640	1420	120	133	169	57

We then used methylene diphenyl diisocyanate to incorporate urethane-based dynamic bonds (MPUr) into these polymers via step-growth polymerization with the PE–OH macromonomers (Figure 1b). Using each of the synthesized PE–OH polymers, we prepared the corresponding HDPE dynamic polymers (PE_1k_MPUr, PE_5k_MPUr, PE_10k_MPUr). In addition, we synthesized a HDPE dynamic polymer with a mixture of all three macromonomers but a similar number-average molecular mass between bonds as PE_5k_MPUr (called PE_5kM_MPUr). We verified successful polymerization of these dynamic HDPE polymers via FTIR by tracking C=O and N–H stretches (Figures 1c,d and S10) and high-temperature SEC (HT-SEC) by tracking increased overall molecular mass (Figure 1e and Table 1). Except for PE_1k_MPUr, which has a much lower overall molecular mass, the other three dynamic HDPE polymers (PE_5k_MPUr, PE_10k_MPUr, PE_5kM_MPUr) have similar *M*_n_ and mass-average molecular masses (*M*_w_) to those of a benchmark HDPE and significantly less than those a benchmark UHMWPE.

To quantitatively describe the effect of bond placement on key material properties, we quantified the bond placement along the backbone using a probability distribution (Figure 2a and Note S1). For each polymer, this bond-to-bond spacing distribution, *p*(*M*_b_), gives the probability density of observing a certain molecular mass (*M*_b_) between two adjacent dynamic bonds. Assuming equal reactivity of all oligomer end groups in our synthesis, this bond spacing distribution is exactly equal to the initial molecular mass distribution of PE–OH backbones. Moreover, we can use *p*(*M*_b_) (Figure 2b) to obtain the corresponding mass-distribution, *w*(*M*_b_) (Figure 2c), and *z*-distribution, *z*(*M*_b_) (Figure 2d), of bond spacings. This is useful since many polymer properties are dictated not by their number-average molecular mass (which treats each chain equally) but by their mass-average (treating each monomer equally) or *z*-average (treating each monomer–monomer interaction equally) molar mass. We hypothesized that instead of the overall chain molar mass, many key properties of these polymers would be primarily determined by these bond spacing distributions. We sought to validate this theory by using the defined bond spacing distributions for our dynamic HDPE polymers to predict key properties, and in the next sections, we demonstrate the advantages of using this approach.

**Figure 2:**
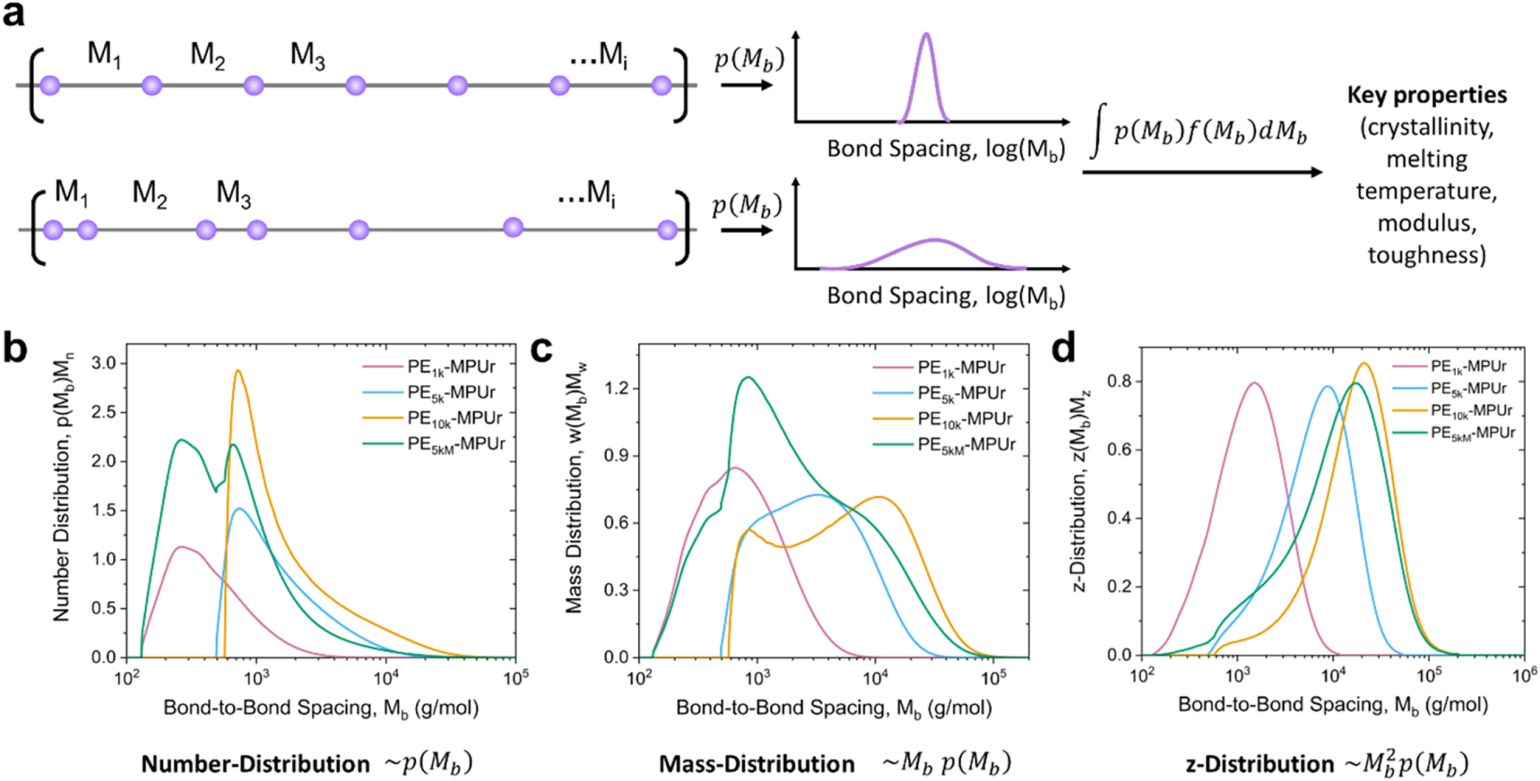
Characterization of bond-to-bond spacing distributions. (a) Defining the bond-to-bond spacing as a probability distribution to predict key properties. Measured number-average (b), mass-average (c), and *z*-average (d) bond spacing distributions for PE_1k_MPUr (pink), PE_5k_MPUr (blue), PE_10k_MPUr (gold), and PE_5kM_MPUr (green).

### Impact of Dynamic Bond Incorporation on HDPE Crystallization

We characterized the thermal transitions of the PE–OH oligomers and the model dynamic HDPE polymers via differential scanning calorimetry (DSC) to estimate the percent crystallinity and peak melting and crystallization temperatures (Figures 3a and S11 and Table 1). We plotted the percent crystallinity versus *M*_w_ of crystallizable segment lengths for the PE–OH polymers, model dynamic polymers, and HDPE and UHMWPE references with a dashed line showing the expected decrease in percent crystallinity with increasing molecular mass of standard polyethylene chains (Figure 3b).^37^ There is a decrease in percent crystallinity with dynamic bond incorporation (Table 1), which decreases in magnitude with increasing spacing between the bonds (which corresponds to lower bond concentration).

**Figure 3:**
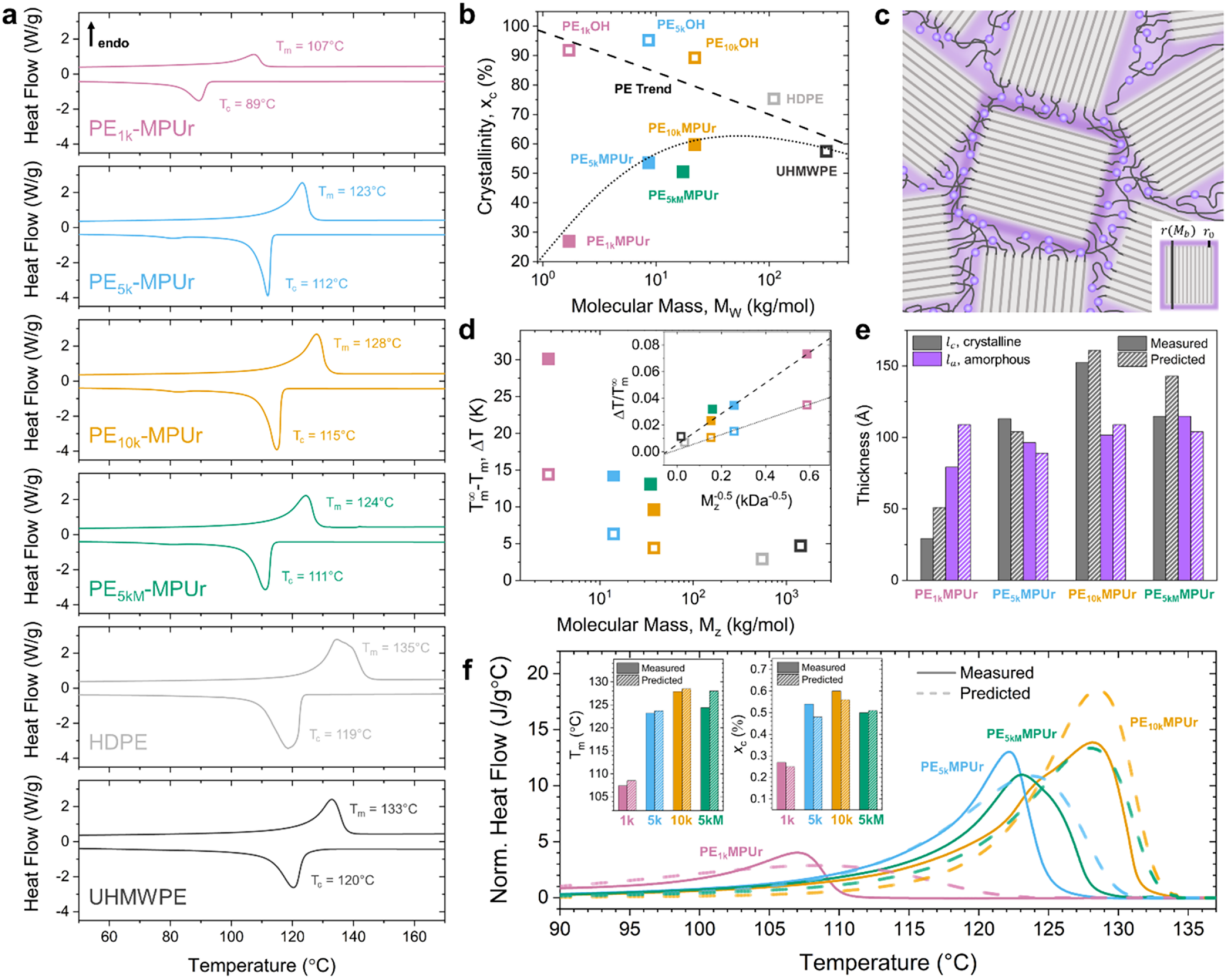
Crystallization of dynamic HDPE polymers. (a) Heat flow from DSC measured, from top to bottom, for PE_1k_MPUr (pink), PE_5k_MPUr (blue), PE_10k_MPUr (gold), PE_5kM_MPUr (green), HDPE (light gray), and UHMWPE (dark gray). Temperatures corresponding to the peak melting (*T*_m_) and crystallization (*T*_c_) rates are marked, endotherm direction is up. (b) Percent crystallinity (*x*_c_) versus molecular mass of crystallizable segments for PE_1k_OH (pink, open), PE_5k_OH (blue, open), PE_10k_OH (gold, open), PE_1k_MPUr (pink, solid), PE_5k_MPUr (blue, solid), PE_10k_MPUr (gold, solid), PE_5kM_MPUr (green, solid), HDPE (light gray, open), and UHMWPE (dark gray, open). The dark gray dashed line represents the trend for unmodified HDPE.^37^ The light gray dotted line shows the predicted values for monodisperse samples from eqs 1 and 2. (c) Schematic representation of dynamic HDPE polymers, showing the dynamic bonds (purple spheres) excluded from the crystal lamellae (light gray) into the amorphous regime. Inset shows the dimensions of the crystal lamellae, *r*(*M*_b_), and the dynamic bond size, *r*_0_. (d) *T*_m_ depression versus molecular mass of crystallizable segments (same legend as b). Inset shows data replotted to extract the change in lamellae surface energy from the slope, with fits shown for the dynamic HDPE polymers (dashed line) and the dihydroxy-terminated HDPE oligomers (dotted line). (e) Measurements of lamellae thickness (*l*_c_) and amorphous thickness (*l*_a_) from SAXS (solid darker) and predicted from the bond spacing distributions (striped lighter). (f) Comparison of experimentally measured heating rate and mass normalized heat flows (solid lines) to predicted heat flows based on bond spacing distributions (dashed) for PE_1k_MPUr (pink, solid), PE_5k_MPUr (blue, solid), PE_10k_MPUr (gold, solid), and PE_5kM_MPUr (green, solid). Inset shows measured (solid darker) and predicted (striped lighter) values for *T*_m_ (left) and *x*_c_ (right).

Previous reports have shown that even small functional groups such as esters or sulfates are excluded from the crystal lamellae.^34,35^ Based on this work and the bulky nature of the MPUr group used here, we hypothesized that all the dynamic bonds would be excluded from the crystal lamellae into the amorphous phase (Figure 3c). Thus, the distribution of bond-to-bond spacings (Figure 2b–d) is also the upper limit of the distribution of crystallizable segment lengths within each polymer chain. We estimated the decrease in crystallinity in the model dynamic HDPE polymers by considering the contribution from each crystallizable segment independently

1 $$x_{c}\left(M_{b}\right)=x_{c}^{0}\left(M_{b}\right)f_{u}\left(M_{b}\right)$$

where *x*_c_^0^(*M*_b_) is the percent crystallinity of a standard PE chain of length *M*_b_, *x*_c_(*M*_b_) is the percent crystallinity after adding the dynamic bond, and *f*_u_(*M*_b_) is the fraction of that segment that is undisturbed by the addition of the dynamic bond and able to crystallize. Since the dynamic bond is excluded from the lamellae, we considered that these excluded dynamic bonds would create an amorphous shell surrounding the crystalline lamellae, which disturbs a section of the backbone on order of the size of the dynamic bond (Figure 3c). This allowed us to estimate *f*_u_(*M*_b_) by

2 $$f_{u}\left(M_{b}\right)=\frac{{\left(r\left(M_{b}\right)-2r_{0}\right)}^{3}}{{\left(r\left(M_{b}\right)\right)}^{3}}$$

where *r*(*M*_b_) = *b̅M*_b_^1/2^ is the expected end-to-end distance for an ideal chain with mass-normalized statistical segment length *b̅*, and *r*_0_ = *M*_bond_^1/3^ρ_bond_^–1/3^ *N*_A_^–1/3^ is the size of the dynamic bond, where *M*_bond_ = 250.25 g/mol, ρ_bond_ = 1.23 g/cm^3^ (from small molecule), and *N*_A_ is Avogadro’s constant.

The predicted *x*_c_ values are shown via the dotted line in Figure 3b. This prediction uses no fitted parameters, only the molar mass (weight-averaged since contributions to crystallinity are per monomer) and density of the dynamic bonding unit, providing strong support that the underlying determinant of percent crystallinity is the bond-to-bond spacing distribution and that these bonds are excluded from the lamellae into the surrounding amorphous region.

We also considered the effect of bond-to-bond spacing on melting temperature. Plotting the measured depression in the melting temperature from the equilibrium melting temperature of HDPE^38^ (*T*_m_^∞^ = 137.5 °C) for each sample versus the *z*-average molar mass of crystallizable segment lengths (since heat flow is dominated by the longest chains with the most monomer–monomer interactions), we see a decrease in melting temperature with decreasing segment length (Figure 3d). Approximating the lamellae thickness as *r*(*M*_b_), we estimated the surface energy penalty difference (γ) before and after dynamic bond incorporation (Figure 3d,inset) using the Gibbs–Thomson equation

3 $$\frac{T_{m}^{\infty }-T_{m}}{T_{m}^{\infty }}=\frac{\gamma }{\mathrm{b̅}{\Delta H}_{V}^{\infty }}{M_{b}}^{-0.5}$$

where ${\Delta H}_{V}^{\infty }={2.95\times 10}^{8}\;J/m^{3}$ is enthalpy of crystallization for HDPE at infinite crystal size, and *b̅* = 1.12 Å/Da^0.5^ is the mass-normalized statistical segment length of HDPE.^39^ This leads to estimated surface energy penalties of γ_OH_ = 0.059 J/m^2^ and γ_bond_ = 0.12 J/m^2^, or a doubling of γ upon bond incorporation at the lamellae surface, consistent with the more bulky and polar nature of the dynamic bond.

To further confirm this analysis, we conducted small-angle X-ray scattering (SAXS, Figure S12) to measure differences in the long period (*L*) of the dynamic HDPE polymers and extracted the average lamellar (*l*_c_) and amorphous layer (*l*_a_) thicknesses by approximating the linear crystallinity (*l*_c_/*L*) with the bulk crystallinity from DSC (Figure 3e, *l*_c_ = *x*_c_ *L*).^40^ The above Gibbs–Thomson analysis suggested that the lamellae thickness *l*_c_ was approximately equal to *r*(*M*_b_). Using the measured bond spacing distributions, we estimated the average *l*_c_ and *l*_a_ for each polymer and observed good agreement with the experimental results (Figure 3e and Note S2).

Finally, we noted that eqs 1 and 3 clearly define *x*_c_ and *T*_m_ as functions of *M*_b_ (Figure S13) and proceeded to combine these relationships with the measured bond spacing distributions to directly estimate the heat flow (normalized by heating rate and mass) expected from melting the sample during DSC (Note S3). Figure 3f shows the theoretical predictions (dashed lines) overlaid over experimental measurements (solid lines). The predicted heat flows clearly capture the general trends observed, with increasing peak melting temperature from PE_1k_MPUr, PE_5k_MPUr, PE_5kM_MPUr, and PE_10k_MPUr. Moreover, this is achieved without fitted parameters (though we use experimental data to estimate γ_bond_ via Figure 3d) and minimal kinetic information, which would result in deviations from equilibrium crystallization behavior.

PE_5kM_MPUr consistently falls off the predicted trendlines for *x*_c_ and *T*_m_ and exhibits the largest difference between predicted and experimental heat flows. Given that these assumptions assume each *M*_b_ contributes independently of other segments, there is no included penalty for a large variance in bond-to-bond spacing, which would certainly frustrate lamellae formation and packing, with the largest such effect observed in PE_5kM_MPUr which has a mixed bond spacing distribution. Indeed, we observe that PE_5kM_MPUr exhibits both lower *x*_c_ and *T*_m_ than anticipated, leading to over prediction of *x*_c_, *T*_m_, and *l*_c_. Importantly, our analysis emphasizes the governing role of the bond spacing distribution in determining key aspects of crystallization, even when bond concentration is held approximately constant, as is the case when comparing PE_5k_MPUr and PE_5kM_MPUr.

### Impact of Bond Placement on Bond Association and Ordering

We next conducted variable-temperature FTIR to measure changes in hydrogen bonding as a function of temperature for each dynamic HDPE polymer during heating (Figures 4a and S14). Based on previous polyurethane research, we assigned the free and hydrogen-bonded NH stretches to the peaks at 3440 cm^–1^ and 3350 cm^–1^, respectively.^41^ The hydrogen-bonded NH stretch is known to have a higher molar absorptivity than the free NH stretch (ε_HB_ > ε_free_), and this difference must be accounted for to accurately determine the relative fraction of hydrogen-bonded units. By tracking changes in the combined peak area with temperature, we directly estimated the molar absorptivity ratio between the hydrogen-bonded and free NH stretches in our polymers $\left(\frac{\varepsilon_{\mathrm{HB}}}{\varepsilon_{\mathrm{free}}}\right)$ to be 3.7 ± 0.4 (Figure S15 and Note S4), which is in line with previously reported values for urethanes between 3 to 4.^42–44^ Figure 4b plots the resulting fraction of associated NH groups as a function of temperature for each polymer. There is a clear increase in bond association when the polymers crystallize. This suggests that crystallization promotes hydrogen bond formation, as opposed to a competing mechanism that limits bond association. We attribute this increase to the exclusion of the bonds into the amorphous phase, which increases their effective concentration. This is supported by the fact that (1) the increase in bond association occurs near the melting temperature of each polymer, and (2) PE_10k_MPUr, which has the highest percent crystallinity, exhibits the largest increase in bond association, while PE_1k_MPUr, which has the lowest percent crystallinity, exhibits the smallest increase in bond association.

**Figure 4:**
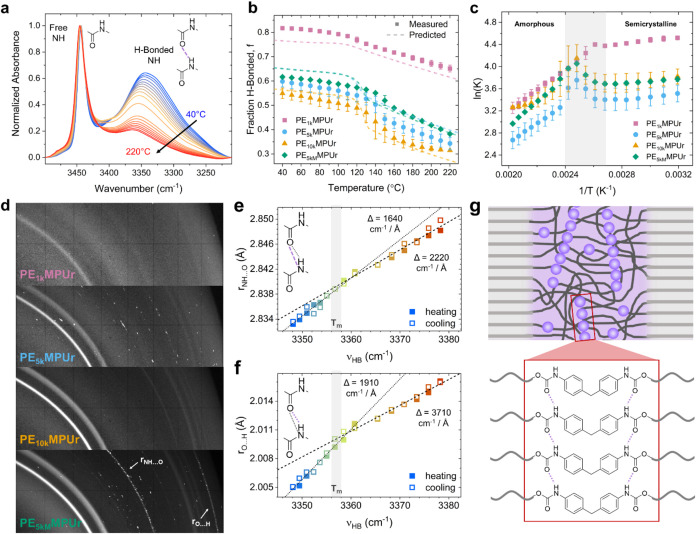
Bond association and ordering in dynamic HDPE polymers. (a) Representative normalized FTIR absorbance of the free and hydrogen-bonded NH stretch from 40 °C (blue, top) to 220 °C (red, bottom) in 10 °C increments (from PE_5k_MPUr). (b) Fraction of hydrogen-bonded NH groups versus temperature for PE_1k_MPUr (pink, squares), PE_5k_MPUr (blue, circles), PE_10k_MPUr (gold, triangles), and PE_5kM_MPUr (green, diamonds). Error bars show standard deviation (*n* = 2). Dashed lines show predicted values calculated using the bond spacing distribution for each polymer. (c) Arrhenius plot of the equilibrium association constant of MPUr showing different behavior in the amorphous and semicrystalline regimes, with the transition regime shaded in gray. (d) Reconstructed 2D WAXS images for PE_1k_MPUr, PE_5k_MPUr, PE_10k_MPUr, and PE_5kM_MPUr (top to bottom) showing the maximum intensity value for each pixel across the full temperature sweep. Arrows mark the rings corresponding to the NH···O and O···H hydrogen bond distances. (e) NH···O hydrogen bond distance and (f), O···H hydrogen bond distance versus FTIR peak position of the hydrogen-bonded NH stretch during heating (solid) and cooling (open) between 60 °C (blue) and 200 °C (red) with 10 °C steps of PE_5kM_MPUr sample. Lines show fits to the semicrystalline regime (dotted) and amorphous regime (dashed). (g) Schematic showing the stacking of MPUr dynamic bonds in the amorphous phase between lamellae. Zoomed region shows the chemical structure of the MPUr stacks, with adjacent bonds connected by intermolecular hydrogen bonding.

Based on this data, we approximated the equilibrium assembly of these bonds within the polymer network using a noncooperative, supramolecular stacking mechanism that has been shown to occur in amorphous systems (Note S5).^29,45,46^ The fraction of associated units formed (*f*) is related to the equilibrium constant (*K*) and total concentration (*C*_T_) by

4 $$K\left(T\right)=\frac{1}{C_{T}\left(T\right)}\left[\frac{f\left(T\right)}{{\left(1-f\left(T\right)\right)}^{2}}\right]$$

In a fully amorphous, homogeneous system, *C*_T_ is independent of temperature; however, in the semicrystalline system, the effective bond concentration in the amorphous phase increases with increasing crystallinity, as dynamic bonds are excluded from the crystal lamellae, which implies

5 $$C_{T}\left(T\right)=\frac{\phi }{1-x_{c}\left(T\right)}$$

where $\phi =\frac{M_{\mathrm{bond}}}{M_{\mathrm{bond}}+M_{n}}$ is the bulk mass fraction of the dynamic bond. Plotting the experimentally measured *K* values on an Arrhenius plot (Figure 4c), we extracted separate values for the enthalpy (Δ*H*) and entropy (Δ*S)* changes for bond association in both the amorphous and semicrystalline regimes (Figure S16 and Note S5). We then estimated the fraction of associated bonds as a function of temperature, *f*(*T*), using the bond-to-bond spacing distribution to determine *x*_c_(*T*) based on the relationships in Figure 3b. Figure 4b shows good agreement between the predicted and measured amount of hydrogen bonding and correctly captures the jump-like increase in association driven by crystallization. Currently, these predictions ignore any effects of bond placement on the underlying bond association mechanism (i.e., Δ*H* and Δ*S* values). Deviations from this assumption could arise from an entropic penalty for creating a network from otherwise free chains as well as penalties from chain stretching required to maximize bond formation, which are unlikely to be independent of bond spacing.

We also conducted wide-angle X-ray scattering (WAXS) on all four dynamic HDPE polymers (Figure S17). In addition to the standard peaks for orthorhombic HDPE crystals, we observed additional peaks (Figure S18). Moreover, on observing the 2D images, we noticed that these peaks appeared as a series of spots, analogous to single-crystal diffraction patterns as well as long-range ordering in block copolymers, albeit at a different length scale.^47,48^ Upon melting the HDPE crystals, these spots remained present, continuously appearing and disappearing during a temperature heat/cool/heat ramp (Videos S1, S2, S3, and S4 and Figure S19), suggesting thermally active rotation and reorganization. We reconstructed new 2D WAXS images, keeping the maximum intensity values across the entire temperature sweep (Figure 4d), as well as only in the amorphous or crystalline regions (Figure S20). Of all the dynamic HDPE polymers, PE_5kM_MPUr exhibited the highest intensity of such spots, at *q* = 2.25 Å^–1^ and *q* = 3.25 Å^–1^ which form two pronounced rings along with additional ring-shaped collections of spots.

Based on previous studies of MPUr in polyurethanes and small molecule crystal analogues, we assigned the two pronounced rings in Figure 4d to the NH···O hydrogen bond distance (*r*_NH···O_) and O···H (*r*_O···H_) hydrogen bond distance between a stacked dimer of MPUr units, with Bragg values of 2.84 Å and 2.01 Å, respectively.^49–51^ Assuming an N–H bond length of 1.0 Å, this gives average in-plane angles of θ_NHO_ = 139°, θ_HNO_ = 28°, and θ_NOH_ = 17°, which are similar to theoretical studies on MPUr in polyurethanes in a stacked dimer configuration.^52^ Moreover, since the shift in the hydrogen-bonded NH stretch is known to correlate with the X–Y hydrogen bonding distance,^53^ we plotted the change in hydrogen bond length for both *r*_NH···O_ and *r*_O···H_ versus the peak frequency of the hydrogen bonded NH stretch from FTIR (ν_HB_) (Figures 4e,f and S21–22 and Note S6). We found a linear relationship that differs slightly in the semicrystalline versus amorphous regime. The frequency shift per angstrom change in hydrogen bond length, were (1640 and 2220) cm^–1^ Å^–1^ for *r*_NH···O_ and (1910 and 3710) cm^–1^ Å^–1^ for *r*_O···H_ in the semicrystalline and amorphous regions, respectively. These values are similar to previous measurements relating the FTIR stretching frequency to hydrogen bond length, and are consistent with the change in the equilibrium constant for bond association between the amorphous and crystalline state (Figure 4c).^53–55^

These observations provide an explanation for the observed spots in the WAXS pattern—long-range ordering of stacked MPUr units with larger grain sizes of 20 nm to 40 nm (estimated by Scherrer analysis), depicted in Figure 4g, that manifest as a spotty 2D pattern (Figure 4g).^56^ PE_5kM_MPUr, which exhibits a unique combination of short and long bond spacings, is able to accommodate the formation of this long-range hydrogen bonding better than any of its individual components, suggesting an interplay of enhanced ordering from dynamic bonds linked by short bond spacings that are free to align when connected by longer backbone segments.

### Impact of Dynamic Bond Incorporation on Mechanical Properties

We next characterized the change in mechanical properties based on the dynamic bond spacing distribution. Rheological temperature ramps (Figure 5a) show a clear transition in the storage modulus (*G*′) of the HDPE dynamic polymers from semicrystalline-dominated behavior (at low temperatures) to dynamic bond-dominated (at high temperatures), which can be seen when comparing plots of percent crystallinity versus temperature from DSC (Figure S23). Unlike HDPE, which does not show a plateau in *G*′ at higher temperatures (since it lacks dynamic bonds and has limited entanglements), all the dynamic HDPE polymers show a plateau at higher temperatures in both *G*′ and tan δ (Figure S24), demonstrating stable formation of a dynamic bonding network even after the melting transition of HDPE. This is consistent with the persistence of the stacked hydrogen-bonding peaks in WAXS at high temperature (Figure S19). UHMWPE, on the other hand, does exhibit a *G*′ plateau, even though it does not have any dynamic bonds, due to the large number of entanglements between its chains. As the bond concentration increases, the magnitude of the *G*′ plateau increases (PE_1k_MPUr > PE_5k_MPUr > PE_10k_MPUr). Interestingly, however, PE_5kM_MPUr has a higher *G*′ plateau than all of them, including PE_1k_MPUr despite having five times fewer dynamic bonds. In addition, PE_5kM_MPUr has a higher *G*′ than PE_5k_MPUr at low temperatures despite having a lower overall crystallinity. We attribute the enhanced modulus in both cases to the formation of long-range stacks of dynamic bonds (present at all temperatures) as suggested by the above WAXS analysis, which serves to reinforce the amorphous matrix and mimic the highly entangled UHMWPE system. Importantly, despite their low tan δ at high temperatures, the dynamic HDPE polymers can still be readily processed into films during melt pressing, by transiently breaking the dynamic interactions. Above 180 °C, all of the HDPE dynamic polymers start to show a decrease in *G*′, which we attribute to the hydrogen bonding network beginning to dissociate, as supported by the FTIR and WAXS data (Figure S21).

**Figure 5:**
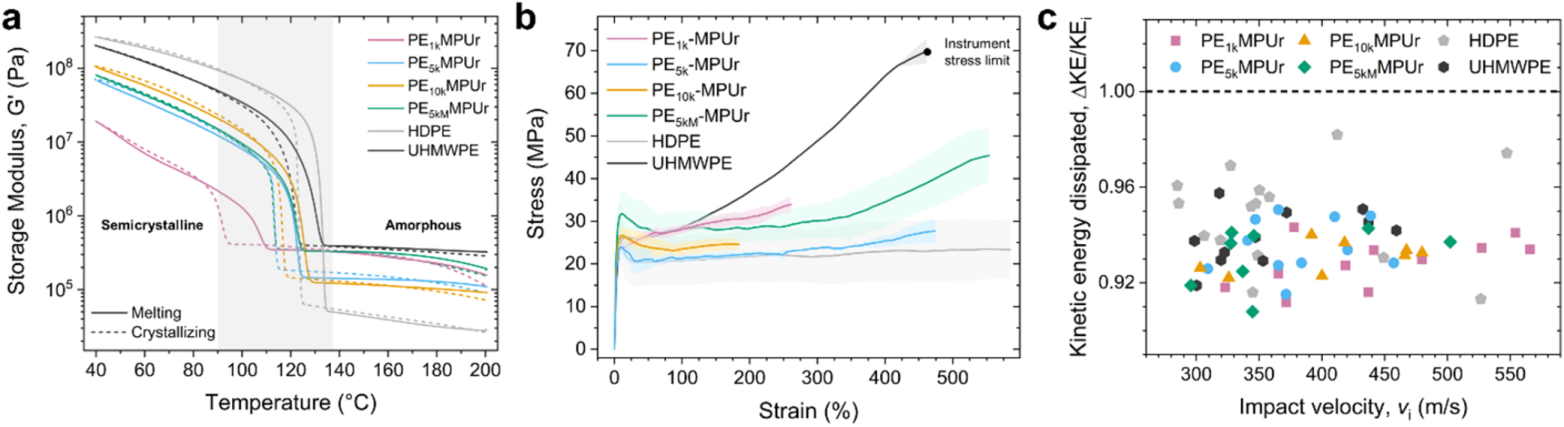
Mechanical properties of dynamic HDPE polymers. (a) Storage moduli for PE_1k_MPUr (pink), PE_5k_MPUr (blue), PE_10k_MPUr (gold), PE_5kM_MPUr (green), HDPE (light gray), and UHMWPE (dark gray) during melting (solid lines) and crystallization (dashed lines). Transition regime marked in gray. (b) Stress–strain curves for PE_1k_MPUr (pink), PE_5k_MPUr (blue), PE_10k_MPUr (gold), PE_5kM_MPUr (green), HDPE (light gray), and UHMWPE (dark gray) measured at room temperature. Shaded areas show standard deviation between samples (*n* = 3). Black dot represents the maximum allowable stress on the instrument. (c) Plot of the kinetic energy loss parameter (ΔKE/KE*_i_*) as a function of impact velocity for PE_1k_MPUr (pink, square), PE_5k_MPUr (blue, circle), PE_10k_MPUr (gold, triangle), PE_5kM_MPUr (green, diamond), HDPE (light gray, pentagon), and UHMWPE (dark gray, hexagon) extracted using microballistic impact testing. Each symbol corresponds to one impact test.

These results were further supported by tensile testing in which PE_5kM_MPUr exhibited the best combination of strain-hardening and extensibility, with properties in-between HDPE and UHMWPE (Figure 5b). PE_1k_MPUr showed increased strain-hardening and tensile strength compared to HDPE, despite having a chain *M*_w_ over 10 times lower (Table 1), though it had lower overall extensibility. Conversely, both PE_5k_MPUr and PE_10k_MPUr had similar tensile strength compared to HDPE, though PE_10k_MPUr also had lower extensibility. We attributed this performance to the reduction of total crystallinity with the addition of dynamic bonds, without a sufficient strengthening of the amorphous phase as observed in PE_1k_MPUr. Nonetheless, PE_5kM_MPUr which possesses both short and long bond-to-bond spacings due to its broader bond spacing distribution (Figure 2c), exhibited the best performance, maintaining both the strain-hardening observed in PE_1k_MPUr and the extensibility achieved in PE_5k_MPUr (Figure 5b), once again consistent with the formation of ordered dynamic bond stacks that reinforce the amorphous matrix. In addition, all the polymers had similar Young’s modulus values except for PE_5kM_MPUr (PE_1k_MPUr: 0.76 ± 0.05 GPa, PE_5k_MPUr: 0.71 ± 0.07 GPa, PE_10k_MPUr: 0.78 ± 0.06 GPa, PE_5kM_MPUr: 0.94 ± 0.14 GPa), which was noticeably higher (though this trend is different from the shear moduli trend at room temperature). Both the rheology and tensile strain data suggest that the hydrogen bonds in PE_5kM_MPUr strengthen the amorphous phase connecting crystals. While the mechanical mechanism behind this strengthening is not completely clear, one potential explanation is that the dynamic bonds broaden the size and shape of the rigid amorphous phase (i.e., noncrystalline monomers with restricted mobility).^57,58^ This is consistent with the broader yielding behavior of the HDPE dynamic polymers compared to HDPE without any dynamic bonds.^59^

Finally, since HDPE and UHMWPE are often used in applications where high impact resistance is needed, such as lightweight body armor, we used microballistic impact testing to evaluate the impact response of the dynamic HDPE polymers at high strain rates (Figures 5c and S25–S26).^60^ Despite having lower crystallinities, all of the dynamic HDPE polymers exhibited high impact strength comparable to both HDPE and UHMWPE, effectively dissipating ≈94% of the initial projectile kinetic energy with impact speeds from 300 m s^–1^ to 550 m s^–1^.

The approach here demonstrates how controlling dynamic bond placement in HDPE can dramatically influence key properties including crystallization, mechanical properties, and microstructure. Many of these effects are predicted using measured bond-spacing distributions and structure–property relationships based on polymer physics theories and minimal fitting. This represents a promising future mechanism for the design of new upcycled polymers for direct recycling of polyolefin waste and the potential to integrate tunable, dynamic interactions into additional applications spaces such as viscosity modification in polymer processing, self-healing and shape memory behavior, and polymer toughness in biological and extreme environments.

## Experimental Section

### Disclaimer

Certain commercial equipment, instruments, or materials are identified in this paper to specify the experimental procedure adequately. Such identification is not intended to imply recommendation or endorsement by the National Institute of Standards & Technology, nor is it intended to imply that the materials or equipment identified are necessarily the best available for the purpose.

### Materials

All chemicals were purchased from Sigma-Aldrich and used as received without further purification unless otherwise noted. cis-Cyclooctene (95%) was passed through a plug of basic Al_2_O_3_ prior to use. Reference HDPE was kindly donated from a commercial supplier. UHMWPE was obtained from Thermo Scientific (043951-A1).

### Synthesis of Poly­(cyclooctene) (PCO) via Ring-Opening Metathesis Polymerization

This procedure was modified from a previously reported synthesis.^36^

#### PCO_1kDa_

A dry 100 mL round-bottom flask equipped with a stir bar and capped with rubber was backfilled with argon and vacuum was pulled three times. Anhydrous chloroform (5 mL), cyclooctene (11.02 g, 100 mmol, CO), and 1,4-diacetoxy-*cis*-2-butene (2.287 g, 13.283 mmol, CTA) were added to the flask via syringe and sparged with argon for 5 min. The flask was immersed in an oil bath at 50 °C. Grubbs second-generation (G2) catalyst (68 mg, 0.08 mmol, Chemical Abstracts Service (CAS) No. 246047–72–3) was added via syringe as a solution in anhydrous-degassed chloroform (1 mL) and then rinsed with anhydrous-degassed chloroform (1 mL). The setup was reacted at 50 °C for 20 h.

#### PCO_5kDa_

A dry 100 mL round-bottom flask equipped with a stir bar and capped with rubber was backfilled with argon and vacuum was pulled three times. Anhydrous chloroform (5 mL), cyclooctene (11.02 g, 100 mmol, CO), and 1,4-diacetoxy-*cis*-2-butene (0.492 g, 2.85 mmol, CTA) were added to the flask via syringe and sparged with argon for 5 min. The flask was immersed in an oil bath at 50 °C. Grubbs second-generation (G2) catalyst (64 mg, 0.08 mmol, Chemical Abstracts Service (CAS) No. 246047–72–3) was added via syringe as a solution in anhydrous-degassed chloroform (1 mL) and then rinsed with anhydrous-degassed chloroform (1 mL). The setup was reacted at 50 °C for 21 h.

#### PCO_10kDa_

A dry 100 mL round-bottom flask equipped with a stir bar and capped with rubber was backfilled with argon and vacuum was pulled three times. Anhydrous chloroform (5 mL), cyclooctene (11.02 g, 100 mmol, CO), and 1,4-diacetoxy-*cis*-2-butene (0.186 g, 1.08 mmol, CTA) were added to the flask via syringe and sparged with argon for 5 min. The flask was immersed in an oil bath at 50 °C. Grubbs second-generation (G2) catalyst (68 mg, 0.08 mmol, Chemical Abstracts Service (CAS) No. 246047–72–3) was added via syringe as a solution in anhydrous-degassed chloroform (1 mL) and then rinsed with anhydrous-degassed chloroform (1 mL). The setup was reacted at 50 °C for 22 h.

#### For All PCO Oligomers

The flask was cooled to room temperature and quenched with ethyl vinyl ether (≈0.1 mL, 1 mmol) to deactivate the G2 catalyst and stirred for an additional 15 min. The flask was cooled to 0 °C. Excess methanol was added to precipitate the polymer as a white powder or wax and stirred for 1 h. The sample was filtered and rinsed with excess methanol. The polymer was redissolved in dichloromethane and ≈100 mg (0.085 mmol) of Pentaerythritol tetrakis­(3,5-di*tert*-butyl-4-hydroxyhydrocinnamate) (Irganox 1010) was added as an antioxidant to prevent cross-linking. Solvent was removed in vacuo to isolate the polymer and store in the freezer at −20 °C.

Estimation of Target Molar Mass: *M*_n,calc_ = (MM of CO) × [CO]/([CTA] + [G2]) + (MM of CTA). MM = molar mass; [] = concentration.

### Synthesis of Hydroxy Telechelic Polyethylene

The procedure was modified from a previously reported synthesis.^36^

#### PE_1kDa_–OH

##### Hydrogenation

PCO_1kDa_ (11 g, 100 mmol of olefin), Irganox (100 mg, 0.1 mmol), and xylene (70 mL) were added together in a 500 mL round-bottom flask. Multiple batches of *p*-toluenesulfonylhydrazide and tributylamine were repeatedly added and refluxed at 140 °C for 6 h each. Batch 1: *p*-toluenesulfonylhydrazide (20 g, 109 mmol), tributylamine (23.1 g, 30 mL, 125 mmol). Batch 2: *p*-toluenesulfonylhydrazide (17 g, 93 mmol), tributylamine (15.4 g, 20 mL, 83 mmol). Batch 3: *p*-toluenesulfonylhydrazide (17 g, 93 mmol), tributylamine (15.4 g, 20 mL, 83 mmol). Total Added: *p*-toluenesulfonylhydrazide (54 g, 295 mmol), tributylamine (54 g, 70 mL, 291 mmol). The mixture was removed from heat and allowed to cool to ≈80 °C, at which point, the polymer was fully precipitated as a white powder by pouring in cold methanol (≈100 mL). The mixture was cooled to room temperature and filtered and rinsed with excess methanol. The sample was dried under vacuum at room temperature.

##### End-Group Deprotection

Hydrogenated PCO_1kDa_ (11 g) was suspended in xylenes (100 mL) and heated to 100 °C to dissolve. Sodium methoxide was added slowly as a solution in methanol (20 mL, 25% by mass sodium methoxide). Excess methanol was distilled and collected and the mixture was reacted at 110 °C for 3 h. The mixture was removed from heat and slightly acidic methanol (1 M, 100 mL) was slowly added to precipitate the product as a white powder. The mixture was cooled to room temperature and stirred for 1 h. The polymer was filtered and rinsed with excess methanol and then dried under vacuum at 80 °C for 24 h.

##### Repeated Hydrogenation (to React Alkenes near End Groups, See Note below)

Mostly hydrogenated and deprotected (from above) PCO_1kDa_ (11 g), xylene (50 mL), *p*-toluenesulfonylhydrazide (20 g, 109 mmol), and tributylamine (15.4 g, 20 mL, 83 mmol) were added together and refluxed at 140 °C for 6 h. The mixture was removed from heat and allowed to cool to ≈80 °C, at which point, the polymer was fully precipitated as a white powder by pouring in cold methanol (≈100 mL). The mixture was cooled to room temperature and filtered and rinsed with excess methanol. The sample was dried under vacuum at room temperature.

#### PE_5kDa_–OH

##### Hydrogenation

PCO_5kDa_ (11 g, 100 mmol of olefin), Irganox (100 mg, 0.1 mmol), and xylene (70 mL) were added together in a 500 mL round-bottom flask. Multiple batches of *p*-toluenesulfonylhydrazide and tributylamine were repeatedly added and refluxed at 140 °C for 6 h each. Batch 1: *p*-toluenesulfonylhydrazide (20 g, 109 mmol), tributylamine (28.6 g, 37 mL, 153 mmol). Batch 2: *p*-toluenesulfonylhydrazide (25 g, 136 mmol), tributylamine (38 g, 50 mL, 208 mmol). Batch 3: *p*-toluenesulfonylhydrazide (20 g, 109 mmol), tributylamine (23.1 g, 30 mL, 124 mmol). Total Added: *p*-toluenesulfonylhydrazide (65 g, 355 mmol), tributylamine (90 g, 70 mL, 486 mmol). The mixture was removed from heat and allowed to cool to ≈80 °C, at which point, the polymer was fully precipitated as a white powder by pouring in cold methanol (≈100 mL). The mixture was cooled to room temperature and filtered and rinsed with excess methanol. The sample was dried under vacuum at room temperature.

##### End-Group Deprotection

Hydrogenated PCO_5kDa_ (11 g) was suspended in xylenes (100 mL) and heated to 115 °C to dissolve. Sodium methoxide was added slowly as a solution in methanol (21 mL, 25% by mass sodium methoxide). Excess methanol was distilled and collected and the mixture was reacted at 115 °C for 3 h. The mixture was removed from heat and slightly acidic methanol (1 M, 100 mL) was slowly added to precipitate the product as a white powder. The mixture was cooled to room temperature and stirred for 1 h. The polymer was filtered and rinsed with excess methanol and then dried under vacuum at 80 °C for 24 h.

##### Repeated Hydrogenation (to React Alkenes near End Groups, See Note below)

Mostly hydrogenated and deprotected (from above) PCO_5kDa_ (7 g), xylene (40 mL), *p*-toluenesulfonylhydrazide (15 g, 82 mmol), and tributylamine (11.6 g, 15 mL, 63 mmol) were added together and refluxed at 140 °C for 6 h. The mixture was removed from heat and allowed to cool to ≈80 °C, at which point, the polymer was fully precipitated as a white powder by pouring in cold methanol (≈100 mL). The mixture was cooled to room temperature and filtered and rinsed with excess methanol. The sample was dried under vacuum at room temperature.

#### PE_10kDa_–OH

##### Hydrogenation

PCO_10kDa_ (11 g, 100 mmol of olefin), Irganox (200 mg, 0.2 mmol), and xylene (50 mL) were added together in a 500 mL round-bottom flask. Multiple batches of *p*-toluenesulfonylhydrazide and tributylamine were repeatedly added and refluxed at 140 °C for 6 h each. Batch 1: *p*-toluenesulfonylhydrazide (20 g, 109 mmol), tributylamine (23.1 g, 30 mL, 124 mmol). Batch 2: *p*-toluenesulfonylhydrazide (20 g, 109 mmol), tributylamine (11.6 g, 15 mL, 62 mmol). Batch 3: *p*-toluenesulfonylhydrazide (20 g, 109 mmol), tributylamine (11.6 g, 15 mL, 62 mmol). Batch 4: *p*-toluenesulfonylhydrazide (20 g, 109 mmol), tributylamine (11.6 g, 15 mL, 62 mmol). Total Added: *p*-toluenesulfonylhydrazide (80 g, 436 mmol), tributylamine (58 g, 60 mL, 311 mmol). The mixture was removed from heat and allowed to cool to ≈80 °C, at which point, the polymer was fully precipitated as a white powder by pouring in cold methanol (≈100 mL). The mixture was cooled to room temperature and filtered and rinsed with excess methanol. The sample was dried under vacuum at room temperature.

##### End-Group Deprotection

Hydrogenated PCO_10kDa_ (11 g) was suspended in xylenes (65 mL) and heated to 125 °C to dissolve. Sodium methoxide was added slowly as a solution in methanol (18 mL, 25% by mass sodium methoxide). Excess methanol was distilled and collected and the mixture was reacted at 125 °C for 3 h. The mixture was removed from heat and slightly acidic methanol (1 M, 100 mL) was slowly added to precipitate the product as a white powder. The mixture was cooled to room temperature and stirred for 1 h. The polymer was filtered and rinsed with excess methanol and then dried under vacuum at 80 °C for 24 h.

##### Repeated Hydrogenation (to React Alkenes near End Groups, See Note below)

Mostly hydrogenated and deprotected (from above) PCO_10kDa_ (11 g), xylene (40 mL), *p*-toluenesulfonylhydrazide (20 g, 109 mmol), and tributylamine (15 g, 20 mL, 83 mmol) were added together and refluxed at 140 °C for 6 h. The mixture was removed from heat and allowed to cool to ≈80 °C, at which point, the polymer was fully precipitated as a white powder by pouring in cold methanol (≈100 mL). The mixture was cooled to room temperature and filtered and rinsed with excess methanol. The sample was dried under vacuum at room temperature.

When checking HT-NMR, a small fraction of unhydrogenated alkenes were detected near the end groups of the chains after end-group deprotection. The hydrogenation step was repeated as described above after conversion to hydroxy end-groups and the residual alkenes were removed.

### Synthesis of Dynamic HDPE Polymers

#### PE_1k_MPUr

PE_1kDa_OH (1.97 g, 1.97 mmol OH end groups) was added to a dry 100 mL round-bottom flask with a stir bar and purged with Argon for 5 min. Under the argon purge, 10 mL of anhydrous tetrachloroethane (TCE) was added and the mixture was stirred at 100 °C for 5 min to dissolve. In a separate dry vial, methylene diphenyl diisocyanate (MDI, 518 mg, 2.07 mmol NCO groups, 1:1.05 OH/NCO ratio) was dissolved into 5 mL of anhydrous TCE. The MDI/TCE solution was added to the flask and then reacted for 1 h at 100 °C. After 1 h, TCE was evaporated under vacuum and the reaction flask was heated to 120 °C and reacted overnight for 16 h. The mixture was heated to 130 °C and reacted for another 24 h. Twenty mL of trichlorobenzene (TCB) and 5 mL of dimethylformamide (DMF) was added to the mixture, which was then heated to 160 °C for 1 h to redissolve product. The polymer was precipitated in methanol and collected via filtration as an off-white, yellow solid. As needed, remaining polymer (stuck on glassware) was redissolved and precipitated to collect. The collected polymer was dried under vacuum at 90 °C for 24 h.

#### PE_5k_MPUr

PE_5kDa_OH (2.05 g, 0.4 mmol OH end groups) was added to a dry 100 mL round-bottom flask with a stir bar and purged with Argon for 5 min. Under the argon purge, 10 mL of anhydrous tetrachloroethane (TCE) was added and the mixture was stirred at 130 °C for 5 min to dissolve. In a separate dry vial, methylene diphenyl diisocyanate (MDI, 107.7 mg, 0.43 mmol NCO groups, 1:1.05 OH/NCO ratio) was dissolved into 2 mL of anhydrous TCE. The MDI/TCE solution was added to the flask and then reacted for 1 h at 130 °C. After 1 h, TCE was evaporated under vacuum and the reaction flask was heated to 140 °C and reacted overnight for 20 h. 40 mL of TCB and 5 mL of DMF was added to the mixture, which was then heated to 160 °C for 1 h to redissolve product. The polymer was precipitated in methanol and collected via filtration as an off-white, yellow solid. As needed, remaining polymer (stuck on glassware) was redissolved and precipitated to collect. The collected polymer was dried under vacuum at 90 °C for 24 h.

#### PE_10k_MPUr

PE_10kDa_OH (2.01 g, 0.2 mmol OH end groups) was added to a dry 100 mL round-bottom flask with a stir bar and purged with Argon for 5 min. Under the argon purge, 10 mL of anhydrous tetrachloroethane (TCE) was added and the mixture was stirred at 130 °C for 5 min to dissolve. In a separate dry vial, methylene diphenyl diisocyanate (MDI, 52.8 mg, 0.211 mmol NCO groups, 1:1.05 OH/NCO ratio) was dissolved into 5 mL of anhydrous TCE. The MDI/TCE solution was added to the flask and then reacted for 1 h at 130 °C. After 1.5 h, TCE was evaporated under vacuum and the reaction flask was heated to 150 °C and reacted overnight for 24 h. 60 mL of TCB and 5 mL of DMF was added to the mixture, which was then heated to 160 °C for 1 h to redissolve product. The polymer was precipitated in methanol and collected via filtration as an off-white, yellow solid. As needed, remaining polymer (stuck on glassware) was redissolved and precipitated to collect. The collected polymer was dried under vacuum at 90 °C for 24 h.

#### PE_5kM_MPUr

PE_1kDa_OH (0.1667 g, 0.1667 mmol OH end groups), PE_5 kDa_OH (0.5 g, 0.1 mmol OH end groups), and PE_10kDa_OH (1.333 g, 0.1333 mmol OH end groups) were added to a dry 100 mL round-bottom flask with a stir bar and purged with Argon for 5 min. Under the argon purge, 10 mL of anhydrous tetrachloroethane (TCE) was added and the mixture was stirred at 140 °C for 5 min to dissolve. In a separate dry vial, methylene diphenyl diisocyanate (MDI, 105.1 mg, 0.42 mmol NCO groups, 1:1.05 OH/NCO ratio) was dissolved into 2 mL of anhydrous TCE. The MDI/TCE solution was added to the flask and then reacted for 1 h at 130 °C. After 1 h, TCE was evaporated under vacuum and the reaction flask remained at 140 °C and reacted overnight for 20 h. 60 mL of TCB and 10 mL of DMF was added to the mixture, which was then heated to 160 °C for 2 h to redissolve product. The polymer was precipitated in methanol and collected via filtration as an off-white, yellow solid. As needed, remaining polymer (stuck on glassware) was redissolved and precipitated to collect. The collected polymer was dried under vacuum at 90 °C for 24 h. For PE_5kM_MPUr, the mass fractions of PE_1k_OH, PE_5k_OH, and PE_10k_OH were 0.083, 0.25, and 0.667, respectively, giving values of *M*_n_ = 4.6 kg/mol, *M*_w_ = 17 kg/mol, and *M*_z_ = 35 kg/mol.

We observed that adding Sn catalysts common in synthesizing polyurethanes caused depolymerization of the urethane bonds at the high reaction temperature, resulting in low final molecular mass.

### Nuclear Magnetic Resonance (NMR) Spectroscopy

Spectra were recorded on a 600 MHz Bruker spectrometer. Chemical shifts were referenced to the residual solvent signal of tetrachloroethane-*d*_2_ (6.0 ppm) or CDCl_3_ (7.26 ppm), as indicated in the spectra captions. The measurement temperature was 100 °C.

### Room-Temperature Size Exclusion Chromatography (RT-SEC)

RT-SEC measurements were conducted on a Tosoh EcoSEC system with differential refractive index (RI) detection coupled to a Wyatt Dawn Heleos II multiangle light scattering detector (18 angles). The separation used tetrahydrofuran (THF) as the eluent at 35 °C, and the stationary phase was a set of two Tosoh mixed pore columns (2× TSKgel GMH_HR_–H). Data were collected using Astra 7, and molar masses were determined based on light scattering data fit to a linear Zimm formalism.^61,62^ A differential refractive index increment (d*n*/d*c*) for polycyclooctene of 0.11 mL/g was used.^63^ Samples were prepared at 2 mg/mL and the flow rate was set to 1 mL/min.

### High-Temperature Size Exclusion Chromatography (HT-SEC)

Molar mass measurements for PE samples were performed on a Tosoh EcoSec HT instrument (HLC08321GPC/HT) with differential refractive index (RI) detection equipped with two Tosoh TSKgel GMHHR-H (S) HT2 columns (13 μm mixed bed, 7.8 mm ID × 30 cm) and one Tosoh TSKgel GMHHR-H (20) HT2 column (20 μm, 7.8 mm ID × 30 cm). Exclusion limit ≈ 4 × 10^8^ g/mol. 1,2,4-trichlorobenzene with 300 ppm (mg/kg) of Irganox 1010 was used as the mobile phase at 160 °C. Samples were dissolved at concentrations between 1 mg/mL to 2 mg/mL for at least 4 h at 160 °C with gentle agitation. Molar mass was calculated using a polystyrene standard curve and corrected using the appropriate Mark–Houwink–Sakurada parameters for polystyrene and polyethylene at 160 °C in 1,2,4-trichlorobenzene (*K*_PS_ = 0.000121; α_PS_ = 0.707; *K*_PE_ = 0.000406; α_PE_ = 0.725; from ASTM D 6474).^64,65^ The following cubic fit was obtained and used (*At* ^3^ + *Bt* ^2^ + *Ct* + *D*; *A* = −0.0010369, *B* = 0.07695, *C* = −2.1600, *D* = 27.038). The uncertainty in molar mass precision obtained by this measurement is ±1.5%. Bond spacing distributions were calculated directly from the respective PE–OH d*w*/dlog *M* curves using Python. The bond spacing distribution for PE_5kM_MPUr was constructed numerically using the respective mass fractions of each backbone type used in synthesis.

### ATR Fourier Transform Infrared (FTIR) Spectroscopy

Spectra were recorded using a Nicolet iS50 FT-IR instrument. Attenuated total reflectance (ATR) measurements were performed at 4 cm^–1^ resolution using a diamond ATR plate. 128 scans were collected. Baseline correction was performed in Python using the asymmetrically reweighted penalized least-squares (ArPLS) in the pybaselines package with a smoothing factor of 10^10^. All absorbance data was normalized to the alkyl C–H stretching peak at 2914 cm^–1^.

### Temperature-Dependent Transmission FTIR Spectroscopy

Spectra were recorded using a Nicolet iS50 FT-IR instrument. Transmission measurements were performed in a temperature-controlled sample cell with CaF_2_ windows (32 mm diameter × 3 mm thickness) at 4 cm^–1^ resolution. 128 scans were collected. Hot-pressed films of each sample were loaded between the CaF_2_ windows and the transmission compartment was thoroughly purged with dry CO_2_-free air. Background spectra of the CaF_2_ windows were collected at 40 °C. Measurements were taken at 10 °C increments from 40 to 220 °C, with a 10 min equilibration time at each temperature step prior to measurement. Data analysis was performed in Python. Baseline correction was performed independently for the NH stretching region (3210 cm^–1^ to 3500 cm^–1^) and the NCO stretching region (2000 cm^–1^ to 3000 cm^–1^), using a linear baseline fit at the region end points. The NH stretching region was fit with two Gaussian peaks, corresponding to the hydrogen-bonded and free NH stretches, which were used to calculate the peak areas. The NCO region was fit with a single Gaussian peak, and at lower temperatures, where a peak could not be clearly identified, no peak was assigned and the peak integration was set to zero.

### Differential Scanning Calorimetry (DSC)

Measurements were performed using a TA Instruments Discovery DSC 2500. Powdered samples (5 mg to 9 mg) were loaded and crimped into aluminum sample pans (T zero). Samples were initially heated to 200 °C and then cooled to −120 or 40 °C and heated to 200 °C for two cycles. Samples were heated and cooled at 10 °C/min with isothermal holds in-between heating and cooling steps of 5 min. Peak melting temperature and degree of crystallinity were calculated from the second heating cycle. Degree of crystallinity values were adjusted based on the total mass fraction of polyethylene for each polymer using a value of the enthalpy of fusion for 100% crystalline polyethylene of Δ*H* = 293.6 J/g.

### Tensile Test Measurements

Room temperature tensile tests were conducted on a TA RSA3 instrument at a Hencky strain rate of 0.01 s^–1^. Samples were prepared by hot-pressing films at 200 °C for 10 min under 4982 N between two Teflon films with 0.1 mm stainless steel shims to control the final thickness. Dog bone samples were cut from hot-pressed films with active middle dimensions of 15 mm × 2 mm × 0.1 mm. Measurements were repeated for at least three trials and all samples were visually checked during and after measurement to ensure uniform strain propagation before failure.

### Rheological Measurements

Dynamic mechanical analyses were conducted using an Ares G2 Rheometer with an 8 mm parallel plate setup in a temperature-controlled convection oven. Samples were cut into 8 mm diameter discs with thicknesses of 0.1 mm to 0.3 mm. Samples were heated to 200 °C under a constant axial compression of 0.1 N. Two preconditioning frequency sweeps were completed at 200 °C at strain of 1% from 100 rad/s to 1 rad/s while maintaining an axial compression of 0.1 N to ensure good contact between the sample and the plates. Temperature ramps at a strain of 1% and 10 rad/s angular frequency were then collected at 5 °C/min from 200 to 40 °C (cooling/crystallizing trace) and from 40 to 200 °C (heating/melting trace) with a 60 s soak time between ramps.

### Microballistic Impact Measurements

Microballistic impact experiments were performed using a laser-induced particle impact test (LIPIT) platform.^66^ Monodisperse silica microprojectiles (20 mm diameter, SiO_2_-R-20.0, microParticles GmbH) were individually launched toward the surface of hot-pressed samples. Each normal impact event was captured by an ultrafast camera (SIMD12, Specialized Imaging Ltd.) with the time between frames ranging from 490 to 660 ns, depending on the microprojectile impact velocity. The impact (*v*_i_) and rebound (*v*_rb_) velocities were calculated by dividing the distance traveled by the microprojectile between two consecutive frames by the time between frames. The energy dissipated by the sample was quantified by the kinetic energy loss parameter, $\frac{\Delta \mathrm{KE}}{{\mathrm{KE}}_{i}}=1-\frac{v_{\mathrm{rb}}^{2}}{v_{i}^{2}}$ .^67^

### SAXS and WAXS Measurements

Simultaneous small-angle X-ray scattering (SAXS) and wide-angle X-ray scattering (WAXS) measurements were carried out at the Functional Materials Beamline (FMB) of the Materials Solutions Network at the Cornell High Energy Synchrotron Source (MSN-C). An X-ray beam energy of 9.7 keV (λ = 1.28 Å) was selected using the 111 reflection of a single-bounce, HPHT diamond monochromator.^68,69^ Harmonic rejection and vertical focusing are provided by a 1 m long, bendable, rhodium-coated monochromatic mirror located approximately 7 m upstream of the experimental hutch at an incident angle of 4 mrad. Experiments were carried out in “bulk-beam” mode, and the monochromatic mirror was used to focus the beam into a spot approximately 0.25 mm^2^ × 0.25 mm^2^ at the sample position, with a total flux of approximately 10^11^ photons/s at 125 mA beam current.

WAXS measurements were obtained using two, two-dimensional (2D) detectors (model Pilatus3 200 K, Dectris AG, Baden-Daettwil, Switzerland) at sample-to-detector distances (SDDs) of approximately 13.0 and 16.2 cm. SAXS measurements were obtained simultaneously with WAXS with a third 2D detector (model Pilatus3 300 K) with a sample-to-detector distance of about 244 cm and equipped with a diode beamstop. Calibration of detector positions and angles was performed by obtaining X-ray powder diffraction patterns from lanthanum hexaboride (LaB_6_) powder (Sigma-Aldrich, St. Louis, MO, USA) and a powder sample of silver behenate sandwiched between Kapton film at the sample position and employing the calibration utility provided as part of the pyFAI software package.^70^

Detector images were azimuthally integrated to produce 1D intensity versus scattering vector, *q* (Å^–1^), over a *q*-range of approximately 0.005 Å^–1^ to 0.11 Å^–1^ for SAXS and 0.67 Å^–1^ to 3.65 Å^–1^ for WAXS. Plots were then corrected for both the incidence beam flux. WAXS peak positions in the 1D patterns were indexed and assigned to different unit cells using a SAXS indexing macro in IGOR Pro (WaveMetrics, Inc.).^71^ WAXS crystallinity was determined using the Ruland method through integration of the PE crystalline and amorphous peaks.^72^

To examine the effect of temperature on the crystallization and crystalline structure in situ, a temperature-controlled Linkam shear cell (CSS450) equipped with Kapton windows was placed in the beam path. Samples were melted at 200 °C, then cooled at 10 °C/min to 60 °C and held for 2 to 5 min. Samples were then reheated to 200 °C at 10 °C/min. SAXS and WAXS were collected every 5 s with a 0.5 s exposure time.

## Supplementary Material











## Data Availability

The data sets and any relevant code generated during and/or analyzed during the current study are available in the NIST Public Data Repository, https://datapub.nist.gov/od/id/mds2-3501. All reported measurement error in this work is one standard deviation of the mean measurement value unless otherwise specified.

## Notes

Detailing explanations and derivations with regards to the bond-to-bond spacing distribution of the urethane dynamic bond functionality and the resultant predictions of lamellae and amorphous thicknesses, and normalized heat flow; Additionally, there are notes on determination of hydrogen bonded N–H groups with regards to differences in molar absorptivity between hydrogen bonded N–H and free N–H from the FTIR data, modeling the noncooperative supramolecular assembly and resultant determination of fraction of hydrogen bonded N–H groups, and bond length calculations determined from WAXS; Characterization of PCO and PE-MPUr polymers, including ^1^H NMR data, ATR-FTIR, transmission FTIR data, molar mass distributions, thermal characterization (DSC and predicted *T*_m_ and %*X*_c_ from the bond-to-bond spacing distributions), hydrogen bonding association parameters, rheological measurements of PE-MPUr polymers (tan delta versus temperature), and microballistic measurements (images of high strain rate impact measurements and resultant rebound velocity versus impact velocity data). X-ray scattering data is also included in SAXS, WAXS, 1D WAXS, and videos of 2D WAXS data of cooling and heating cycles of all PE-MPUr polymers (PDF)
2D WAXS PE-_1k_MPUr (Video S1) (AVI)
2D WAXS PE-_10k_MPUr (Video S2) (AVI)
2D WAXS PE-_5k_MPUr (Video S3) (AVI)
2D WAXS PE-_5kM_MPUr (Video S4) (AVI)
