# Supplementary material for: Tuning High-Density Polyethylene Microstructure and Properties from Known Distributions of Dynamic Bonds
Source: J Am Chem Soc. 2025 Dec 4;147(50):46061–74. doi: 10.1021/jacs.5c13586 (PMC12715785; doi:10.1021/jacs.5c13586)
Supplement: Supplementary file 1 [file ja5c13586_si_001.pdf]

Supplementary information for

Tuning High Density Polyethylene Microstructure and Properties from Known Distributions of Dynamic Bonds

Christopher B. Cooper[1], McKenzie L. Coughlin[1], Polette J. Centellas[1], Aaron A. Burkey[1], Kalman B. Migler[1], Jonathan E. Seppala[1], Edwin P. Chan[1], Chad R. Snyder[1], Sara V. Orski[1*]

[1]Materials Science and Engineering Division, National Institute of Standards and Technology, Gaithersburg, Maryland, 20899 USA

Official contribution of the National Institute of Standards and Technology; not subject to copyright in the United States

Table of Contents *(Please use Ctrl + Click to use embedded hyperlink)*

Supplementary Text (Notes S1-S6) 3

Note S1. Integrations over the bond-to-bond spacing distribution to estimate average properties. 3

Note S2. Prediction of lamellae and amorphous thicknesses from bond-spacing distribution. .. 4

Note S3. Predicting the normalized heat flow from a bond spacing distribution. 4

Note S4. Estimating the difference in molar absorptivity between hydrogen-bonded NH and free NH from experimental data. 6

Note S5. Modeling association as non-cooperative supramolecular assembly and estimating fraction of hydrogen-bonded NH groups. 7

Note S6. Estimation of average NH…O and O…H bond lengths from wide-angle x-ray scattering (WAXS) analysis. 10

Figures S1-S26. 11

Figure S1. Proton nuclear magnetic resonance spectroscopy (^{1}H NMR) of PCO_{1k}. 11

Figure S2. ^{1}H NMR of PCO_{5k}. Measured in Chloroform-d at 25 °C. 12

Figure S3. ^{1}H NMR of PCO_{10k}. Measured in Chloroform-d at 25 °C. 13

Figure S4. Molar mass distribution of PCO measured by room-temperature size-exclusion chromatography (SEC). 14

Figure S5. ^{1}H NMR of $PE_{1k}OH$. 15

Figure S6. ^{1}H NMR of $PE_{5k}OH$. 16

Figure S7. ^{1}H NMR of $PE_{10k}OH$. 17

Figure S8. Attenuated Total Reflection Fourier Transform Infrared Spectra (ATR-FTIR) of telechelic dihydroxy HDPE. 18

Figure S9. Molar mass distribution of telechelic dihydroxy HDPE, from high-temperature size exclusion chromatography (HT-SEC). 19

Figure S10. FTIR of dynamic HDPE polymers. 20

Figure S11. DSC of telechelic dihydroxy HDPE. 21

Figure S12. SAXS of PE_{1k}MPUr, PE_{5k}MPUr, PE_{10k}MPUr, PE_{5kM}MPUr, and HDPE. 22

Figure S13. Predicted melting temperature and percent crystallinity from bond-to-bond spacing. 23

Figure S14. Normalized FTIR absorbance of dynamic HDPE polymers. 24

Figure S15. Estimation of molar absorptivity ratio between hydrogen-bonded and free NH..25

Figure S16. Measured hydrogen bond association parameters. 26

Figure S17. Indexed 1D WAXS data. 27

Figure S18. WAXS of dynamic polymers. 28

Figure S19. 1D WAXS data for PE_{5kM}MPUr. 29

Figure S20. Reconstructed 2D WAXS data. 30

Figure S21. WAXS data for NH…O bond at different temperatures. 31

Figure S22. WAXS data for O…H bond at different temperatures. 32

Figure S23. Percent crystallinity versus temperature extracted from DSC measurements. 33

Figure S24. Tan delta versus temperature from rheological measurements. 34

Figure S25. Images of high-strain rate microballistic impact measurements. 35

Figure S26. Rebound velocity versus impact velocity. 36

Descriptions of Videos (Video S1-S4) **37**

Video S1. 2D WAXS of PE1kMPUr upon melting. 37

Video S2. 2D WAXS of PE10kMPUr upon melting. 37

Video S3. 2D WAXS of PE5kMPUr upon melting. 37

Video S4. 2D WAXS of PE_5kMMPUr upon melting. 37

Supplementary Text (Notes S1-S6)

Note S1. Integrations over the bond-to-bond spacing distribution to estimate average properties.

For each polymer, we defined this bond-to-bond spacing distribution, which gives the probability of observing a certain molecular mass between two adjacent dynamic bonds (M_b) on the polymer chain, $p(M_b)$. Using the defined $p(M_b)$ distribution of bond spacings, $w(M_b)$ and $z(M_b)$ can be found respectively by:

$$w(M_b) = \frac{M_b p(M_b)}{\int dM_b M_b p(M_b)} \tag{S1}$$

$$z(M_b) = \frac{M_b^2 p(M_b)}{\int dM_b M_b^2 p(M_b)} = \frac{M_b w(M_b)}{\int dM_b M_b w(M_b)} \tag{S2}$$

One can readily obtain the M_n, M_w, or M_z from these distributions either by integrating appropriately over $p(M_b)$, $w(M_b)$, and $z(M_b)$:

$$M_n = \int dM_b M_b p(M_b) \tag{S3}$$

$$M_w = \int dM_b M_b w(M_b) \tag{S4}$$

$$M_z = \int dM_b \, M_b z(M_b) \tag{S5}$$

The calculation of M_n, M_w, or M_z is a specific case of calculating an average property relative to the $p(M_b)$, $w(M_b)$, or $z(M_b)$ distributions, respectively. In this case, the property defined by M_b is exactly M_b (i.e., $f(M_b) = M_b$). More generally for any property that can be defined as a function of M_b given by $f(M_b)$, the average of f over the $x(M_b)$ distribution (where $x(M_b) = p(M_b)$, $w(M_b)$, or $z(M_b)$) is given by:

$$\langle f \rangle_x = \int dM_x f(M_b) x(M_b)$$

(S6)

Note S2. Prediction of lamellae and amorphous thicknesses from bond-spacing distribution.

$$\langle l_c \rangle = \frac{\int_0^\infty dM_b l_c(M_b) x_c(M_b) w(M_b)}{\int_0^\infty dM_b x_c(M_b) w(M_b)}$$

(S7)

Where $l_c(M_b)$ is given by:

$$l_c(M_b) = \bar{b} M_b^{\frac{1}{2}}$$

(S8)

Then using the same assumption as used for small angle x-ray scattering (SAXS) data analysis, we define the long-period as:

$$L(M_b) = \frac{l_c(M_b)}{x_c(M_b)}$$

(S9)

Then the average value of the long-period is estimated by:

$$\langle L \rangle = \frac{\int_0^\infty dM_b l_c(M_b) w(M_b)}{\int_0^\infty dM_b x_c(M_b) w(M_b)}$$

(S10)

Finally, we obtain the amorphous thickness by noting:

$$\langle l_a \rangle = \langle L \rangle - \langle l_c \rangle$$

(S11)

Note S3. Predicting the normalized heat flow from a bond spacing distribution.

Combining these relationships with the measured bond-to-bond spacings in **Figure 2**, allows us to directly estimate the mass and heating flow normalized heat flow expected from melting the sample during differential scanning calorimetry (DSC), based on the following equation:

$$Q(T) = \frac{\delta}{\delta T}\left(\int_0^{M(T)} dM_b \, x_c(M_b) \Delta H_m(M_b) w(M_b)\right)$$

(S12)

Where:

$$\Delta H(M_b) = \Delta H_v^\infty \left(\frac{T_m}{T_m^\infty}\right)$$

(S13)

$$M(T) = \left(\frac{\gamma T_m^\infty}{\bar{b}\Delta H_v^\infty (T_m^\infty - T)}\right)^2$$

(S14)

Where ΔH_v^∞ is the enthalpy of fusion of the perfect crystal per unit mass, T_m^∞ is the equilibrium melting temperature of the crystal, γ is the surface energy of the crystal, and $\bar{b}$ is the mass normalized statistical segment length. Note that the integrand of Eq. S12 is dependent on both $w(M_b)$ and $x_c(M_b)$, leading to a double dependance on molar mass, best represented by the z-average.

While **Eq. S12** is numerically straightforward to calculate, it can be readily simplified analytically, by noting that the adjacent integral and derivative transform the variable from molar mass space to temperature space, to obtain:

$$Q(T) = x_c\big(M(T)\big)\Delta H_m\big(M(T)\big)w\big(M(T)\big)\frac{dM}{dT}$$

(S15)

An alternative and more complete derivation of **Eq. S15** can be obtained by first considering the fraction of crystals that melt between T and T + dT given by f(T) and the direct relationship to g(l), the crystal size distribution for thicknesses between l + dl.

$$g(l)dl = f(T)dT$$

(S16)

Then we note that the crystal size distribution can be directly related to the chain molar mass between bonds, M, using h(M), the probability of having a crystal comprising a chain of molar mass M + dM:

$$g(l)dl = h(M)dM$$

(S17)

Which allows us to write:

$$f(T) = h(M)\frac{dM}{dT} = x_c(M)w(M)\frac{dM}{dT}$$

(S18)

Where $x_c(M)$ is the percent crystallinity for a bond-to-bond spacing of M and $w(M)$ is the mass-average probability of observing a bond-to-bond spacing of M.

Then we note that the normalized heat flow over time is related to f(T) by:

$$HF_{norm}(t)dt = \Delta H_m\big(M(T)\big)f(T)dT$$

(S19)

Where we note that the enthalpy of fusion for the crystal $\Delta H_m(M(T))$ is proportional to the melting temperature of the crystal (as given in **Eq. S13**). Then we obtain:

$$HF_{norm}(t) = \beta \Delta H_m\big(M(T)\big) f(T) \tag{S20}$$

Where β is the heating rate given by $\beta = \frac{dT}{dt}$, and HF_{norm} is the mass normalized heat flow (in W/g). Normalizing $HF_{norm}(t)$ by β to obtain $Q(T)$ and substituting in for $f(T)$, we obtain **Eq. S15**, completing the derivation. In addition, noting that

$$\frac{dM}{dT} = \frac{2M(T)}{(T_m^\infty - T)} \tag{S22}$$

Finally, for completeness, we note we can write Q(T) explicitly in T by re-writing **Eq. S15**:

$$\begin{aligned} Q(T) &= \Delta H_v^\infty \left(\frac{T}{T_m^\infty}\right) x_c\big(M(T)\big) w\big(M(T)\big) \frac{2M(T)}{(T_m^\infty - T)} \\ &= \Delta H_v^\infty \left(\frac{T}{T_m^\infty}\right) x_c\big(M(T)\big) w\big(M(T)\big) \frac{2}{(T_m^\infty - T)} \left(\frac{\gamma T_m^\infty}{\bar{b} \Delta H_v^\infty (T_m^\infty - T)}\right)^2 \\ &= x_c\big(M(T)\big) w\big(M(T)\big) \frac{2\gamma^2 T_m^\infty T}{\bar{b}^2 \Delta H_v^\infty (T_m^\infty - T)^3} \\ &= x_c\left(\left(\frac{\gamma T_m^\infty}{\bar{b}\Delta H_v^\infty (T_m^\infty - T)}\right)^2\right) w\left(\left(\frac{\gamma T_m^\infty}{\bar{b}\Delta H_v^\infty (T_m^\infty - T)}\right)^2\right) \frac{2\gamma^2 T_m^\infty T}{\bar{b}^2 \Delta H_v^\infty (T_m^\infty - T)^3} \end{aligned} \tag{S23}$$

Note S4. Estimating the difference in molar absorptivity between hydrogen-bonded NH and free NH from experimental data.

Starting from Beer's Law and noting that the total concentration of NH bonds is the sum of concentrations of the hydrogen bonded and free NH bonds, we obtain:

$$C_{NH} = C_{HB} + C_{free} = \frac{A_{HB}}{\varepsilon_{HB} L} + \frac{A_{free}}{\varepsilon_{free} L} \tag{S12}$$

Where ε_{HB} and ε_{free} are the absorptivities of the bonded and free NH stretch, respectively, and L is the pathlength. Then, assuming that the absorptivities are constant within the temperature range tested,[1] we have:

$$C_{NH} L \varepsilon_{free} = \frac{\varepsilon_{free}}{\varepsilon_{HB}} A_{HB} + A_{free}$$

$$R = \alpha A_{HB} + A_{free} = A_{NH} - (1-\alpha)A_{HB}$$

(S13)

Where $R = C_{NH}L\varepsilon_{free}$ is a constant and $\alpha = \frac{\varepsilon_{free}}{\varepsilon_{HB}}$. Noting R is a constant, we can replace it with the mean values of A_{tot} and A_{HB}:

$$R = \overline{A_{NH}} - (1-\alpha)\overline{A_{HB}}$$

(S14)

Rearranging we obtain:

$$A_{NH} - \overline{A_{NH}} = (1-\alpha)(A_{HB} - \overline{A_{HB}})$$

(S15)

Plotting this for all samples creates a master curve whose slope corresponds directly to the value of α. We obtain $\alpha = 0.27$, which corresponds to $\varepsilon_{HB} = 3.7\varepsilon_{free}$. With a defined α, we can now directly calculate the fraction of bonded NH groups (f) using:

$$f = \frac{A_{HB}}{A_{HB} + \frac{A_{free}}{\alpha}}$$

(S16)

Free NH is rarely observed in most polyurethanes in which NH can hydrogen bond with not only the C=O from other urethanes or esters in the system but also the O of any ether bonds in the backbone, which are common. In our system, the dynamic bond is the only source of heteroatoms (atoms other than C or H) in the system and thus the only places in which hydrogen bonding can occur are the dynamic bond sites, making the NH region more reliable for analysis and significantly enhancing the strength of the free NH peak.

Note S5. Modeling association as non-cooperative supramolecular assembly and estimating fraction of hydrogen-bonded NH groups.

We assumed the equilibrium assembly of the dynamic bonds within the high-density polyethylene (HDPE) dynamic polymer network could be approximated by an isodesmic or free association model for supramolecular assembly with a 1D stacking mechanism. This assumes that the probability of a monomer binding to another monomer or to another i-mer stack is identical. This produces an i-mer distribution analogous to the Flory description for linear condensation, and thus, the number average probability of observing an i-mer follows a geometric distribution:

$$p(A_i) = f^{i-1}(1-f)$$

(S17)

where f is the fraction of hydrogen-bonded NH groups. We note that equivalently, we can describe the concentration of $[A_i]$ at equilibrium, with equilibrium constant K, as:

$$A + A \overset{K}{\rightleftharpoons} A_2\ ;\ A_2 + A \overset{K}{\rightleftharpoons} A_3;\ A_{i-1} + A \overset{K}{\rightleftharpoons} A_i$$

$$[A_i] = K^{i-1}[A]^i$$

(S18)

Analogously, under a geometric distribution, for a total concentration of A given by C_T, we note that:

$$C_T = \sum_{i=0}^{\infty} i[A_i] = [A]_0$$

(S19)

Moreover, we can define the total concentration of individual i-mers (regardless of their respective size) as C_P.

$$C_P = \sum_{i=0}^{\infty} [A_i] = C_T(1-f)$$

(S20)

Then we have that:

$$[A_i] = p(A_i)C_P = C_T(1-f)^2 f^{i-1}$$

(S21)

Combining the equations yields:

$$K = \left(\frac{C_T f^{i-1}(1-f)^2}{{C_T}^i(1-f)^{2i}}\right)^{\frac{1}{i-1}} = \frac{1}{C_T}\frac{f}{(1-f)^2}$$

(S22)

Solving for f and restricting to physically reasonable values between 0 and 1, we obtain:

$$f = 1 + \frac{1-\sqrt{4KC_T+1}}{2KC_T}$$

(S23)

Eq. S23 allows for the estimation of the fraction of hydrogen-bonded NH groups from the product KC_T, which are both functions of temperature. We approximated $C_T(T)$ by:

$$C_T(T) = \frac{\phi}{1 - x_c(T)}$$

(S24)

Where $x_c(T)$ is given by:

$$x_c(T) = x_{c,max} - \int_0^{T(M_b)} dM_b x_c(M_b) w(M_b)$$

(S25)

Where $x_{c,max}$ is the maximum crystallinity obtained from integrating to infinity, such that as $M_b \to \infty, x_c \to 0$. From our experimental results, we found that $K(T)$ could be approximated by considering an amorphous and a semicrystalline regime with respective enthalpy (ΔH) and entropy (ΔS) values for all polymers. To estimate $K(T)$, we interpolated the estimated values based on the normalized fraction of the sample melted at a temperature, T, given by:

$$\bar{x}_m(T) = 1 - \frac{x_c(T)}{x_{c,max}}$$

(S26)

Then we have:

$$K(T) = \exp\left(\frac{\Delta H(T)}{RT} - \frac{\Delta S(T)}{R}\right)$$

(S27)

where:

$$\Delta H(T) = \Delta H_{crystalline} + \bar{x}_m(T)(\Delta H_{amorphous} - \Delta H_{crystalline})$$

(S28)

$$\Delta S(T) = \Delta S_{crystalline} + \bar{x}_m(T)(\Delta S_{amorphous} - \Delta S_{crystalline})$$

(S29)

Where $\Delta H_{crystalline}$ = -2.15 kJ/mol, $\Delta H_{amorphous}$ = -15.1 kJ/mol, $\Delta S_{crystalline}$ = 25.5 J/mol K, $\Delta S_{amorphous}$ = -5.7 J/mol K for all of the dynamic polymers.

Note S6. Estimation of average NH…O and O…H bond lengths from wide-angle x-ray scattering (WAXS) analysis.

From the WAXS data, we approximated the average NH…O bond length and the average O…H bond length using an intensity-weighted average over narrow q ranges of (2.18 to 2.24) $Å^{-1}$ and (3.09 to 3.17) $Å^{-1}$, respectively.

$$\langle r \rangle = \frac{\int_{q_{min}}^{q_{max}} dq \frac{2\pi}{q} I(q)}{\int_{q_{min}}^{q_{max}} dq\, I(q)} \tag{S30}$$

Figures S1-S26.

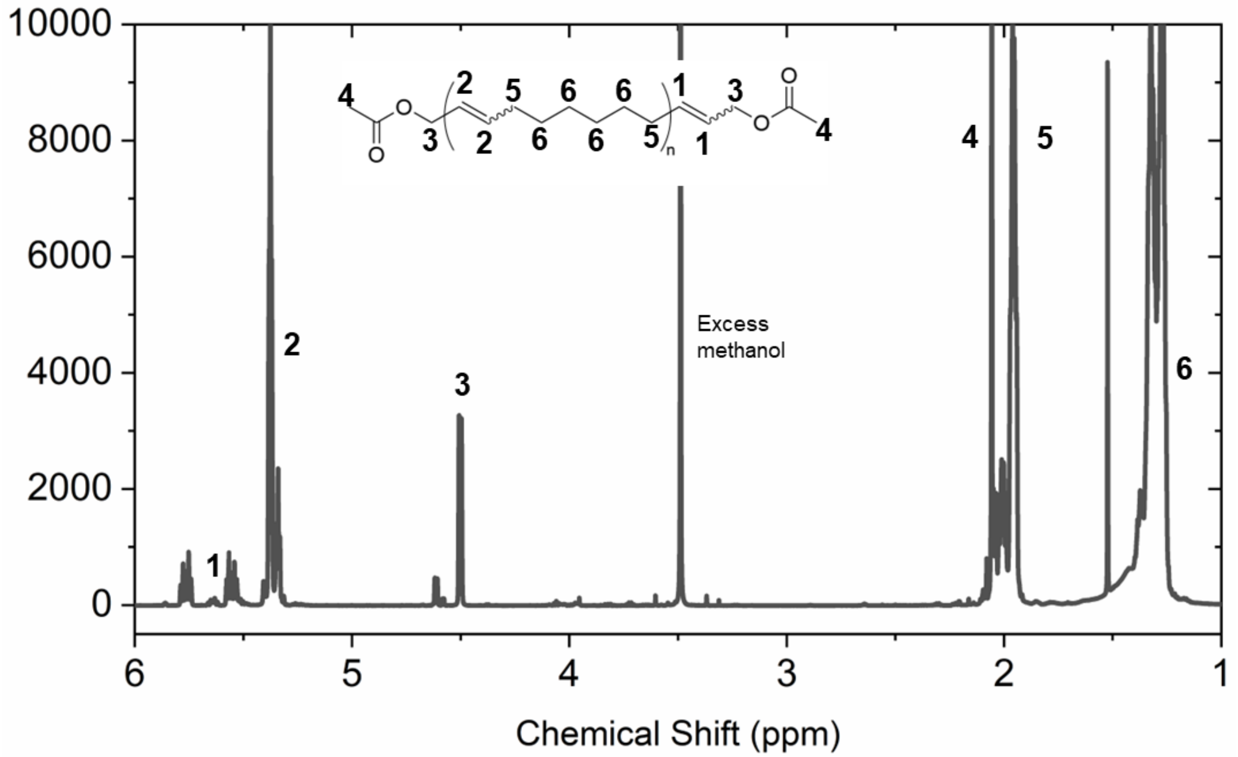

<u>Figure S1. Proton nuclear magnetic resonance spectroscopy (¹H NMR) of PCO$_{1k}$.</u> Measured in Chloroform-*d* at 25 °C. Number of repeat units estimated to be n = 8.2, giving an M_n value of 1.1 kg/mol. Normalized integrals from **1** to **6**: 1.96, 2.09, 17.93, 4.00, 6.34, 36.79, and 80.50.

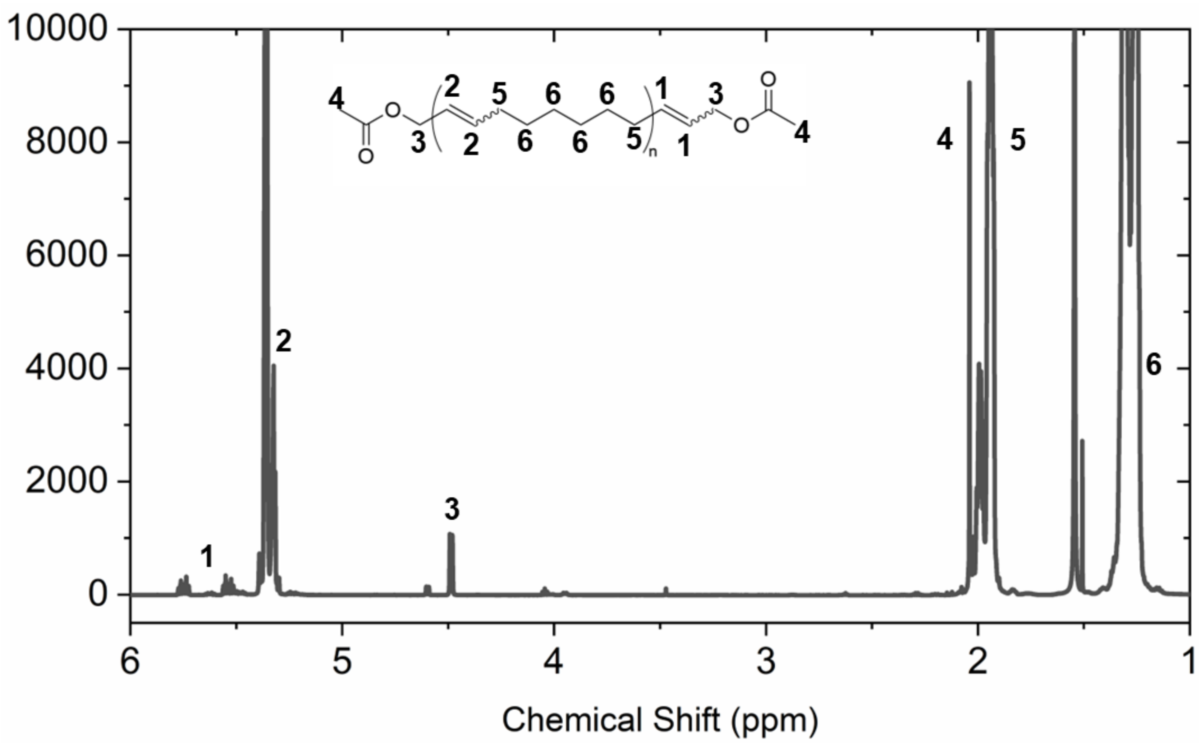

Figure S2. ^{1}H NMR of PCO$_{5k}$. Measured in Chloroform-d at 25 °C. Number of repeat units estimated to be n = 43.4, giving an M_n value of 5.0 **kg/mol**. Normalized integrals from 1 to 6: 1.89, 2.04, 93.69, 4.00, 6.74, 187.97, and 385.67.

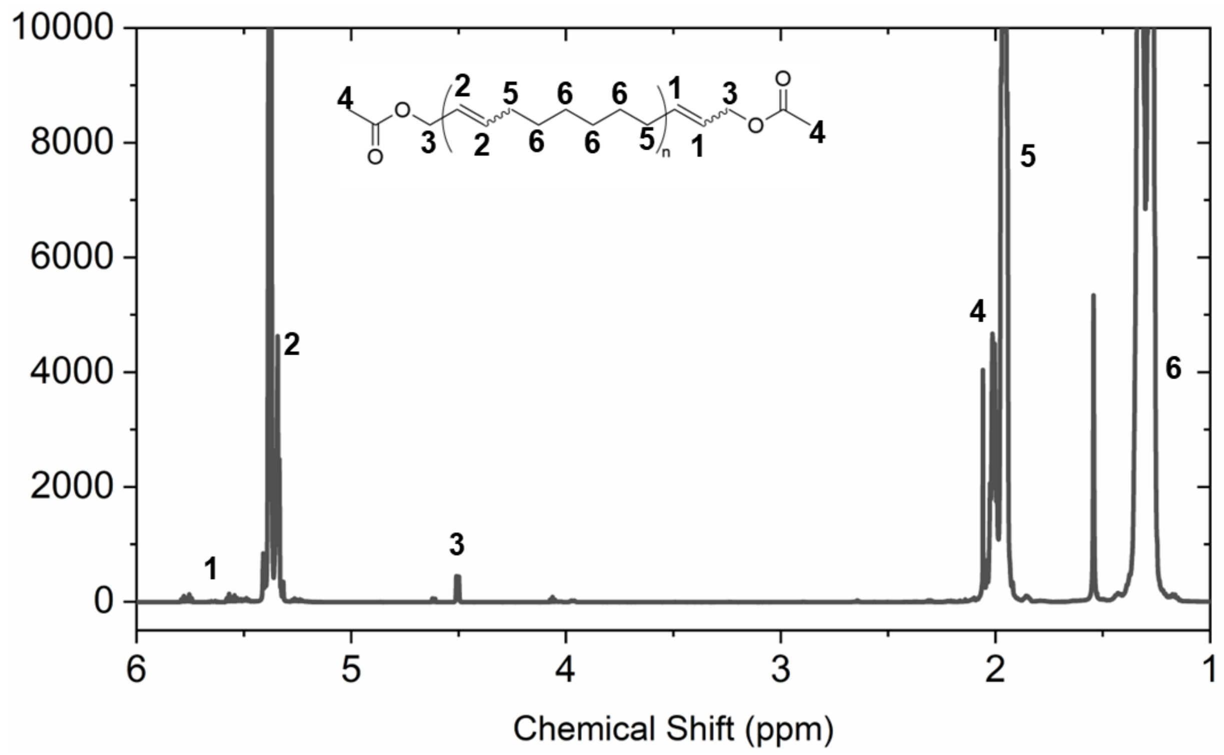

Figure S3. ^{1}H NMR of PCO$_{10k}$. Measured in Chloroform-d at 25 °C. Number of repeat units estimated to be n = 124.6, giving an M_n value of 13.9 kg/mol. Normalized integrals from 1 to 6: 1.78, 1.98, 260.47, 4.00, 6.69, 524.26, and 1063.60.

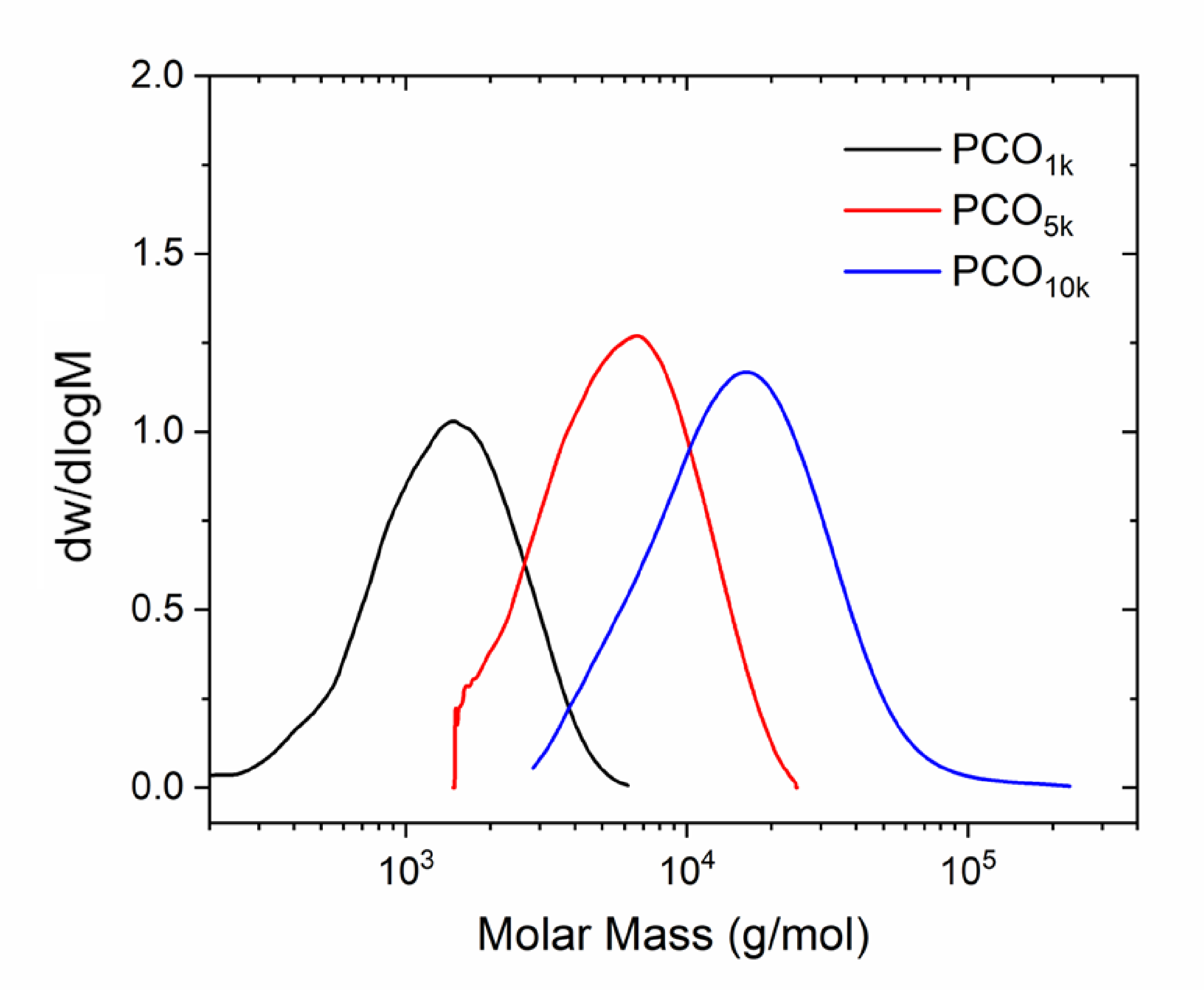

<u>Figure S4. Molar mass distribution of PCO measured by room-temperature size-exclusion chromatography (SEC).</u> Plotted for PCO_{1k} (black, M_n = 1.3 kg/mol, M_w = 2.2 kg/mol), PCO_{5k} (red, M_n = 4.3 kg/mol, M_w = 6.5 kg/mol), and $PCO_{10k}OH$ (blue, M_n = 10.8 kg/mol, M_w = 18.6 kg/mol). Measured in tetrahydrofuran.

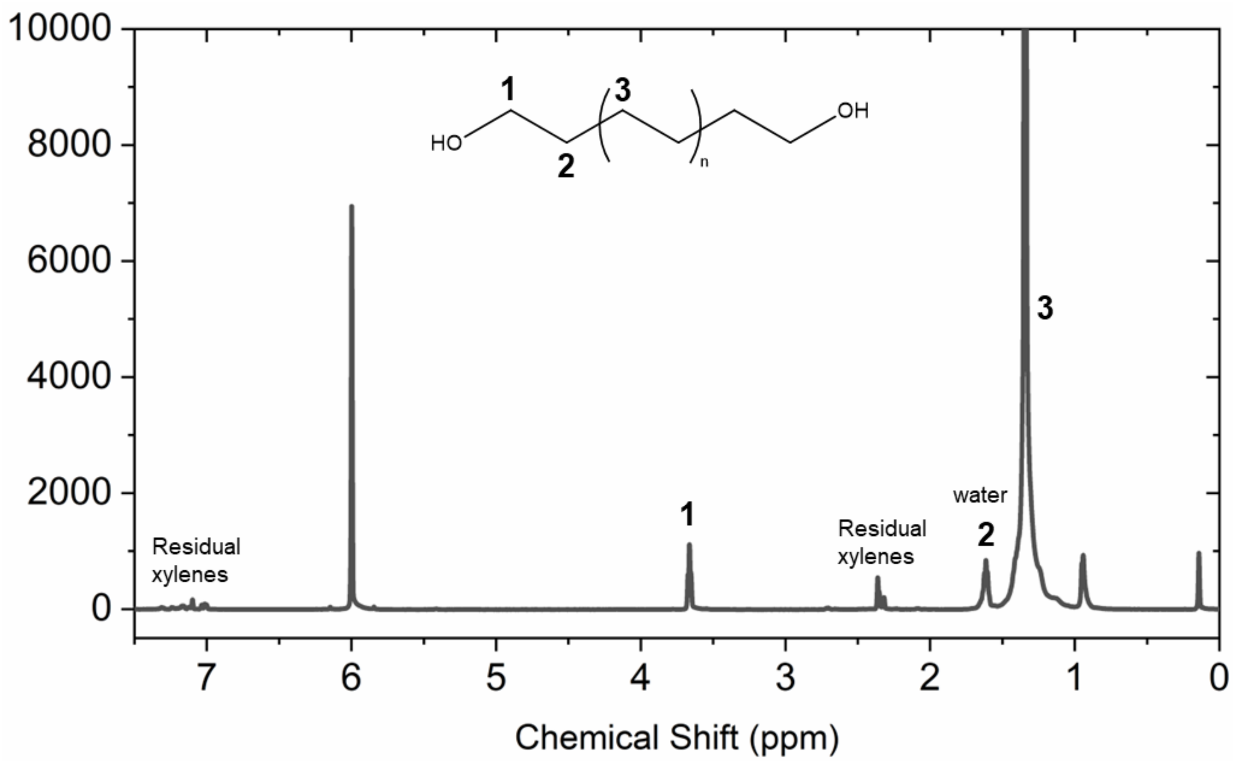

<u>Figure S5. ^{1}H NMR of PE_{1k}OH.</u> Measured in 1,1,2,2-Tetrachloroethane-d_2 at 100 °C. Normalized integrals from **1** to **3**: 4.00, 4.57, and 215.03.

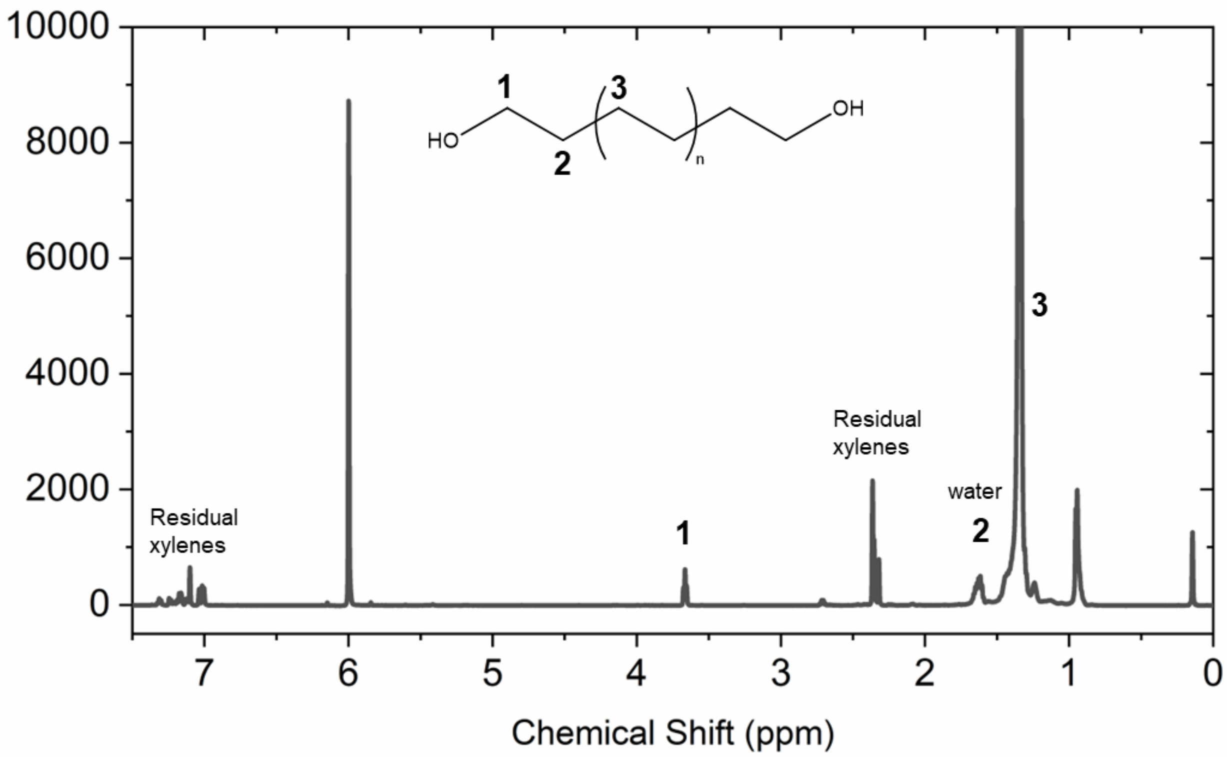

<u>Figure S6. ^{1}H NMR of PE$_{5k}$OH.</u> Measured in 1,1,2,2-Tetrachloroethane-d_2 at 100°C. Normalized integrals from **1** to **3**: 4.00, 8.74, and 766.32.

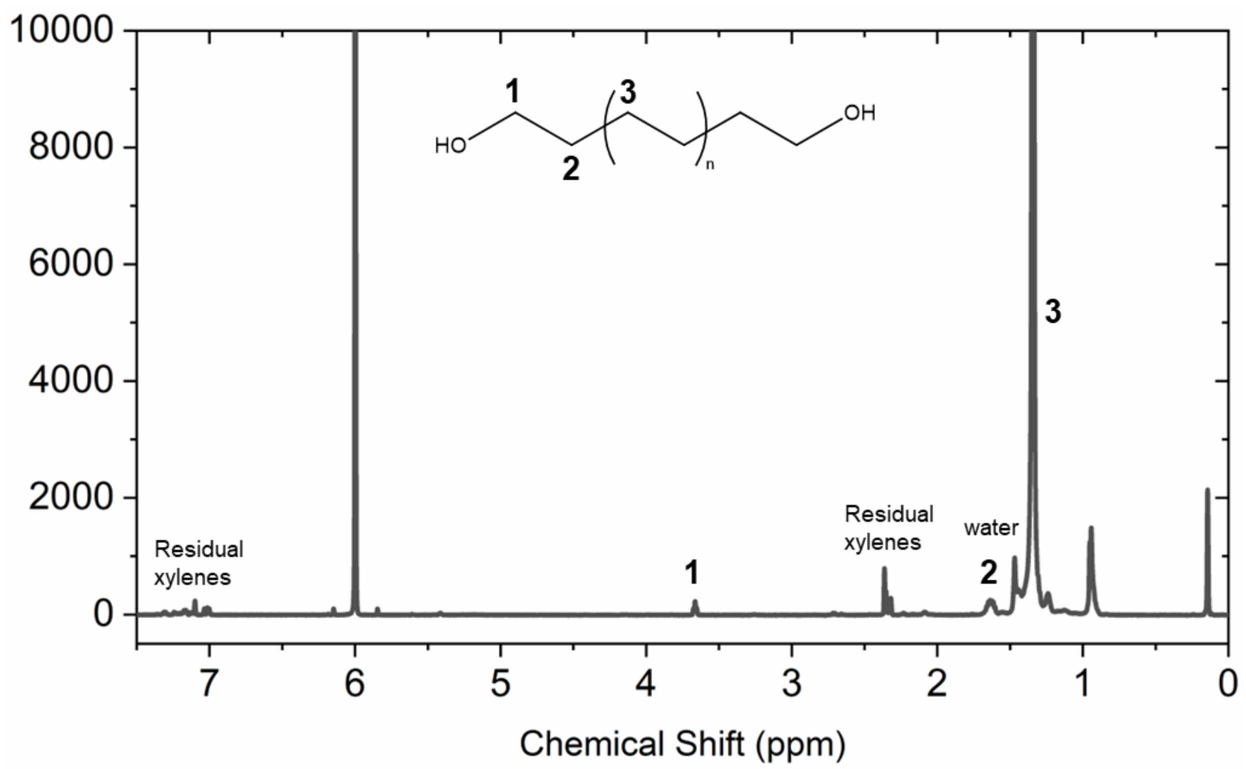

<u>Figure S7. ^{1}H NMR of PE_{10k}OH.</u> Measured in 1,1,2,2-Tetrachloroethane-d_2 at 100°C. Normalized integrals from **1** to **3**: 4.00, 13.70, and 1920.90.

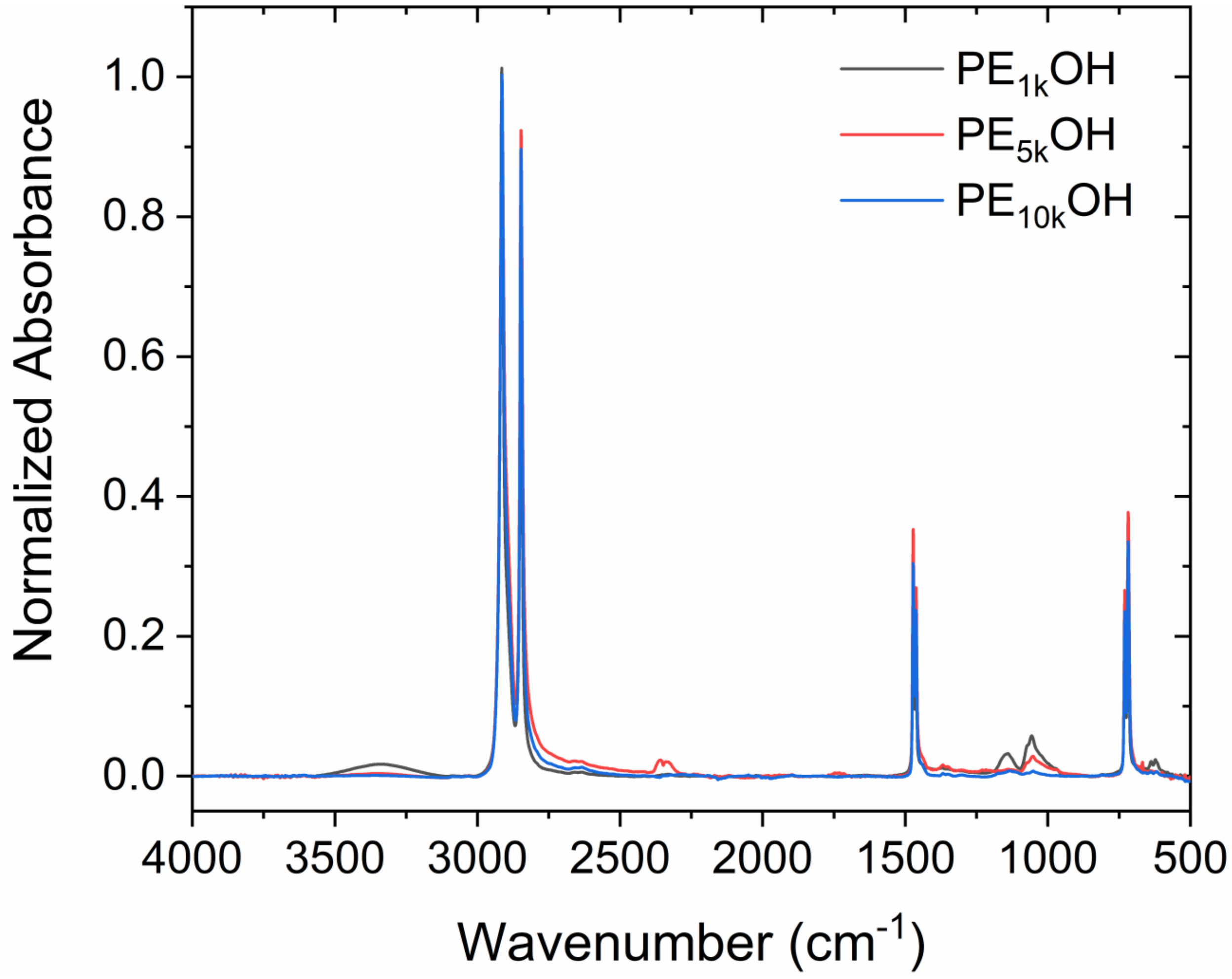

<u>Figure S8. Attenuated Total Reflection Fourier Transform Infrared Spectra (ATR-FTIR) of telechelic dihydroxy HDPE.</u> Plotted for $PE_{1k}OH$ (black), $PE_{5k}OH$ (red), and $PE_{10k}OH$ (blue).

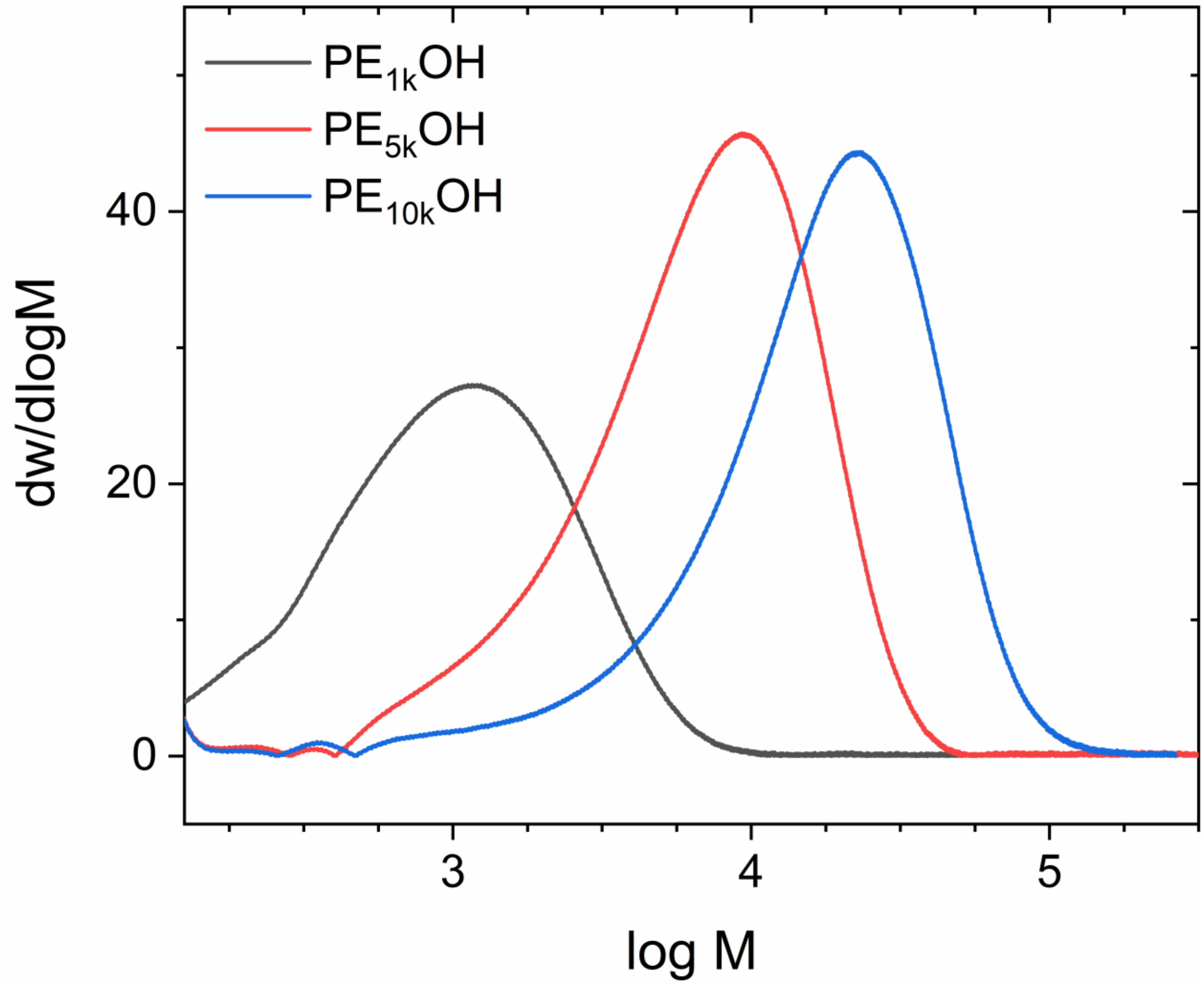

Figure S9. Molar mass distribution of telechelic dihydroxy HDPE, from high-temperature size exclusion chromatography (HT-SEC). Plotted for PE$_{1k}$OH (black), PE$_{5k}$OH (red), and PE$_{10k}$OH (blue) in 1,2,4-trichlorobenzene.

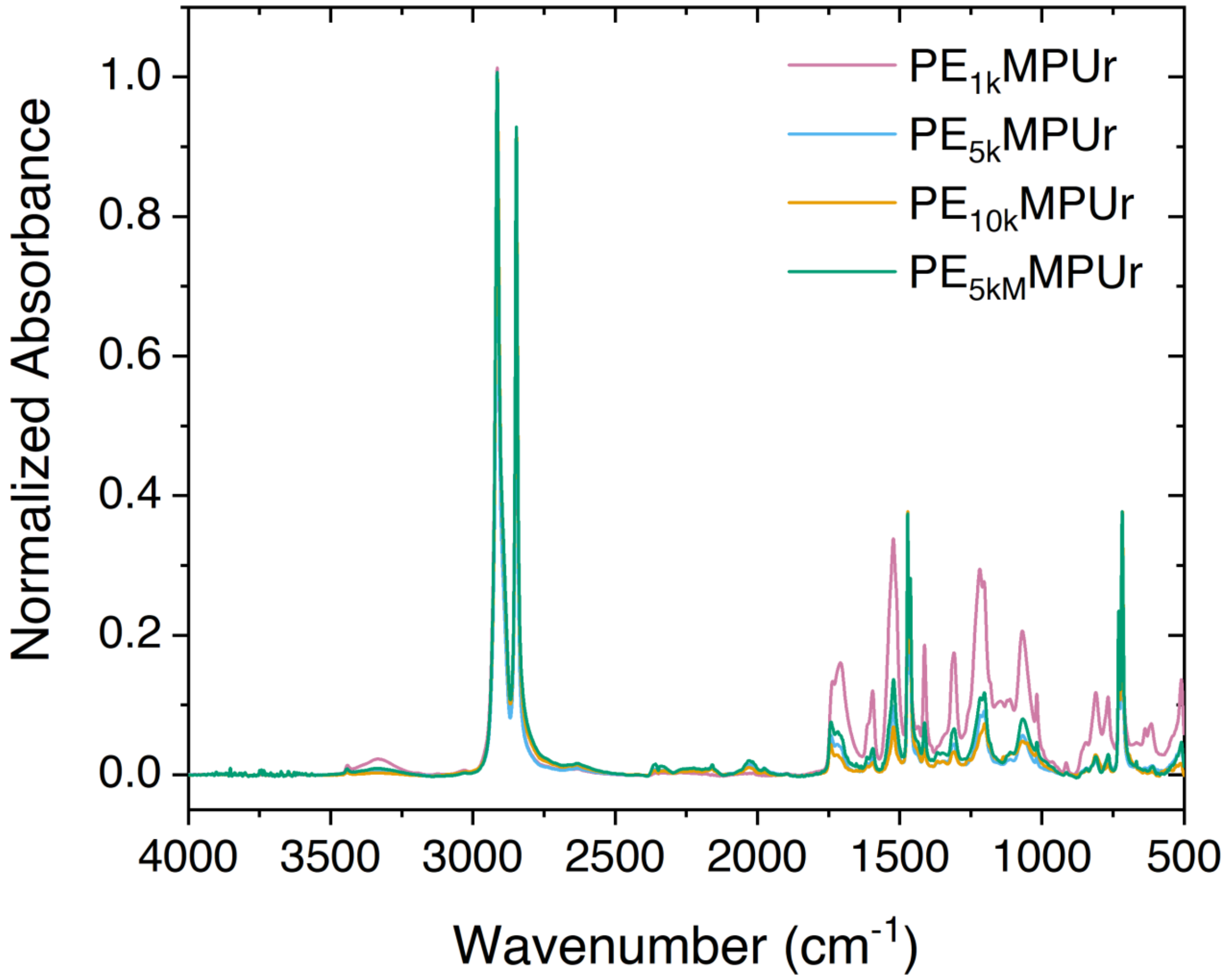

<u>Figure S10. FTIR of dynamic HDPE polymers.</u> Plotted for PE_{1k}MPUr (pink), PE_{5k}MPUr (blue), PE_{10k}MPUr (gold), and PE_{5kM}MPUr (green).

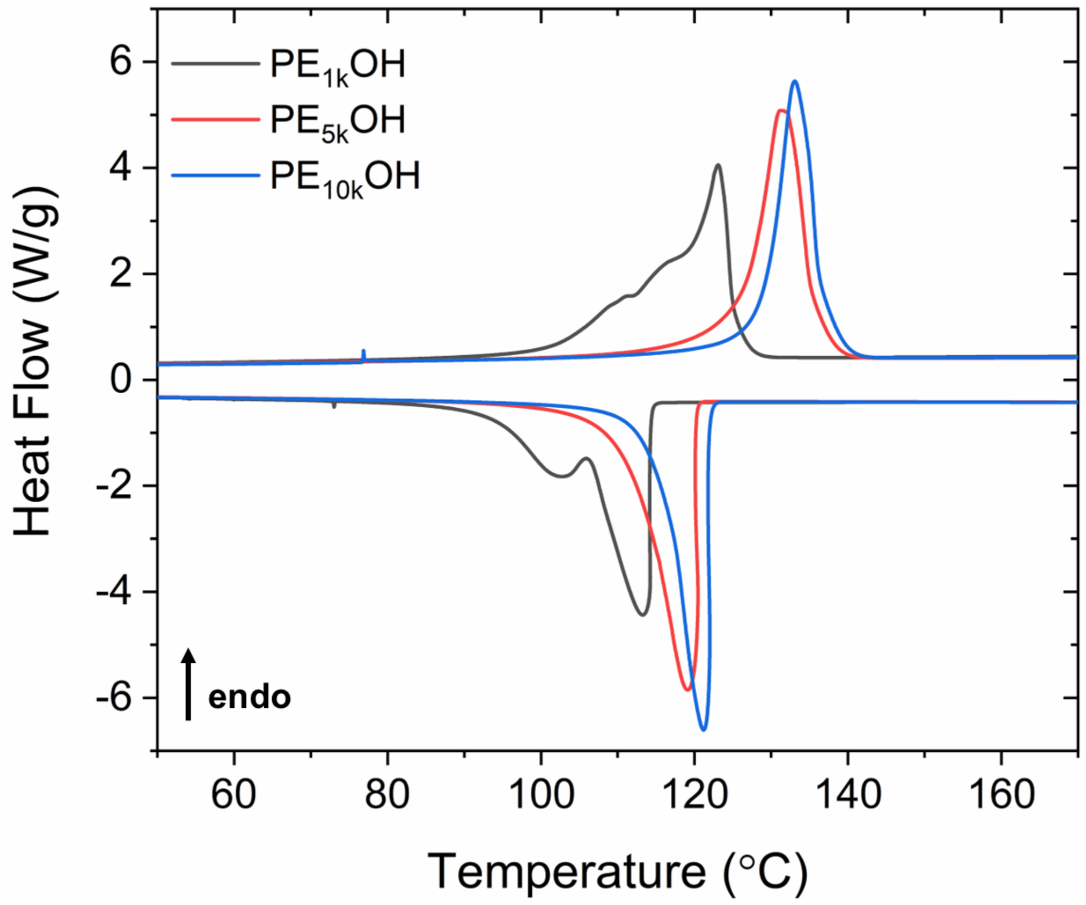

Figure S11. DSC of telechelic dihydroxy HDPE. Plotted for $PE_{1k}OH$ (black), $PE_{5k}OH$ (red), and $PE_{10k}OH$ (blue).

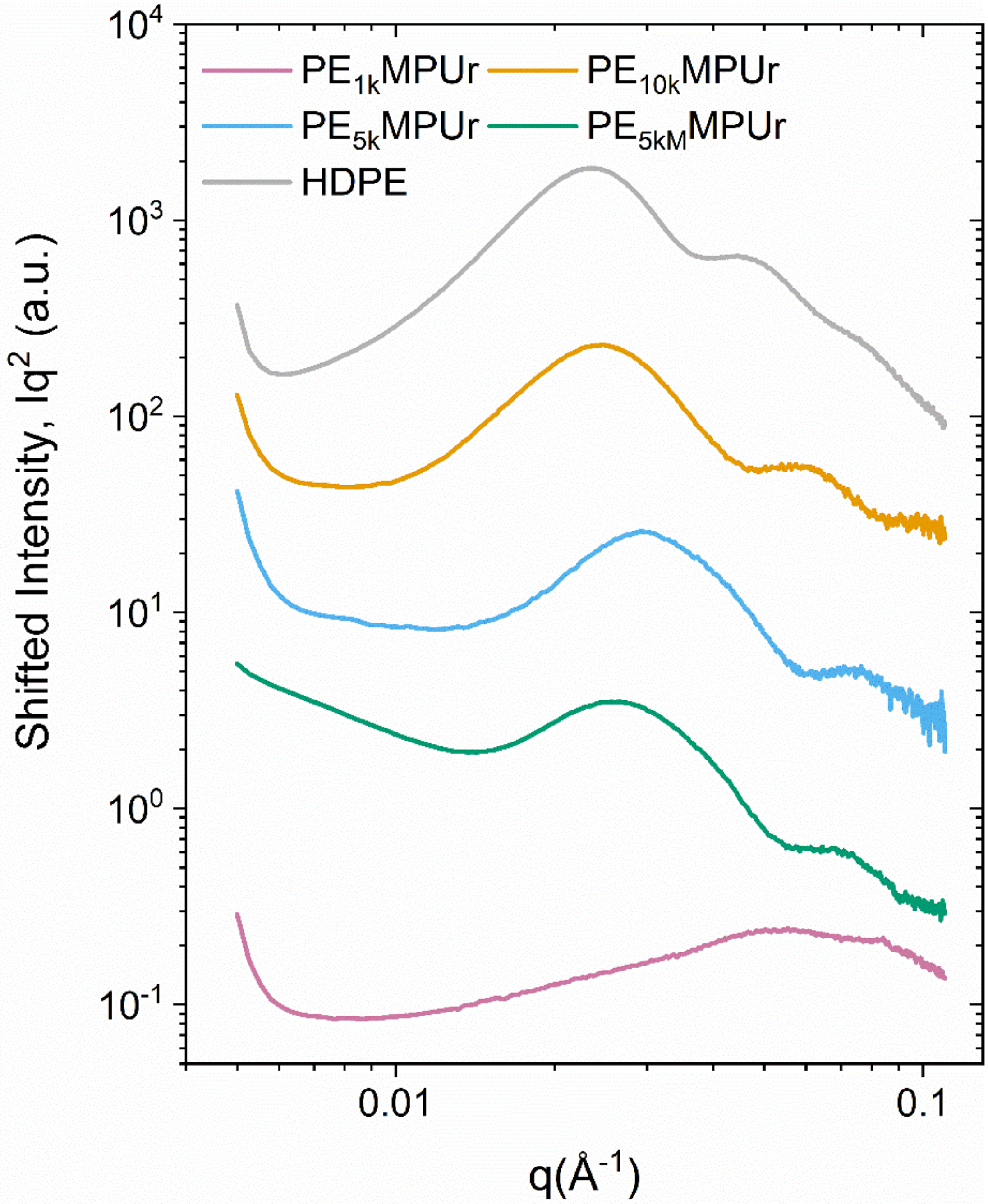

Figure S12. SAXS of PE_{1k}MPUr, PE_{5k}MPUr, PE_{10k}MPUr, PE_{5kM}MPUr, and HDPE. Lorentz-corrected data is plotted with the y-axis shifted for clarity. From top to bottom: HDPE (grey), PE_{10k}MPUr (gold), PE_{5k}MPUr (blue), PE_{5kM}MPUr (green), and PE_{1k}MPUr (pink).

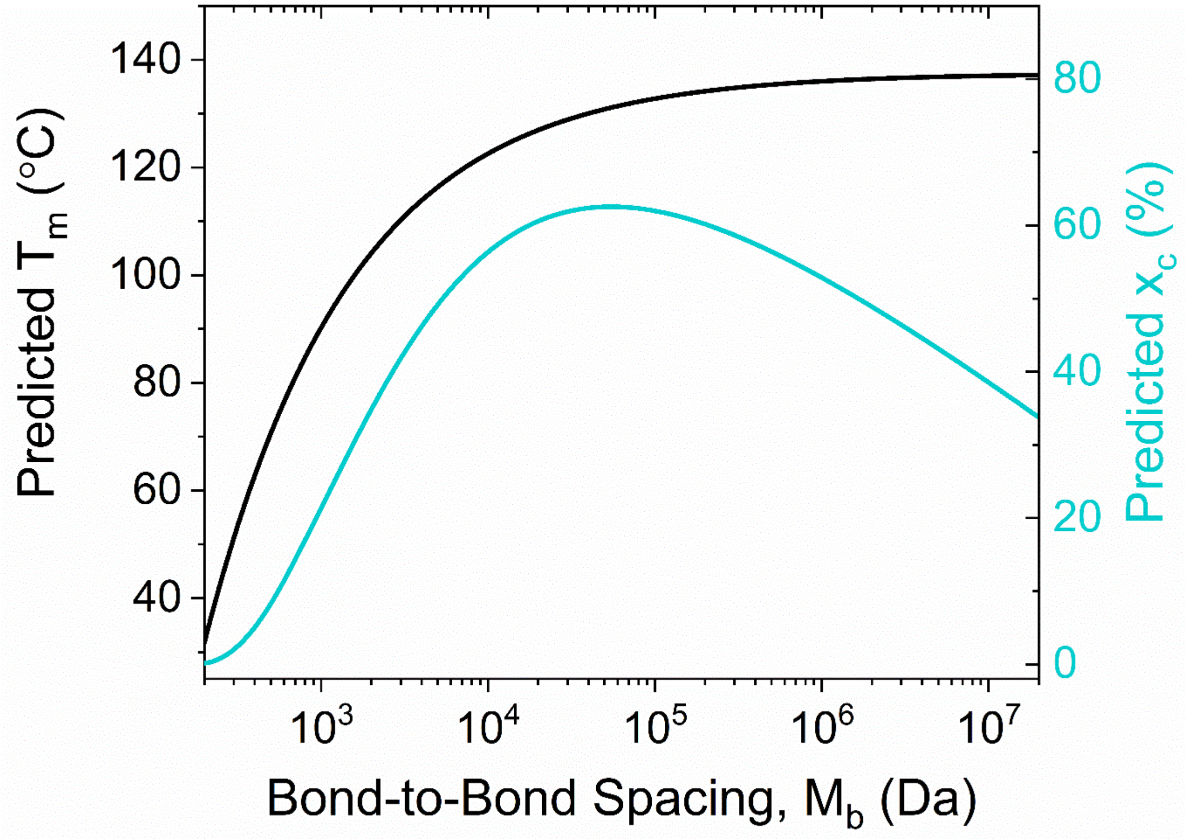

Figure S13. Predicted melting temperature and percent crystallinity from bond-to-bond spacing. Melting temperature (top, black, left axis) and percent crystallinity (bottom, cyan, right axis) are plotted with the same x-axis of bond-to-bond spacing.

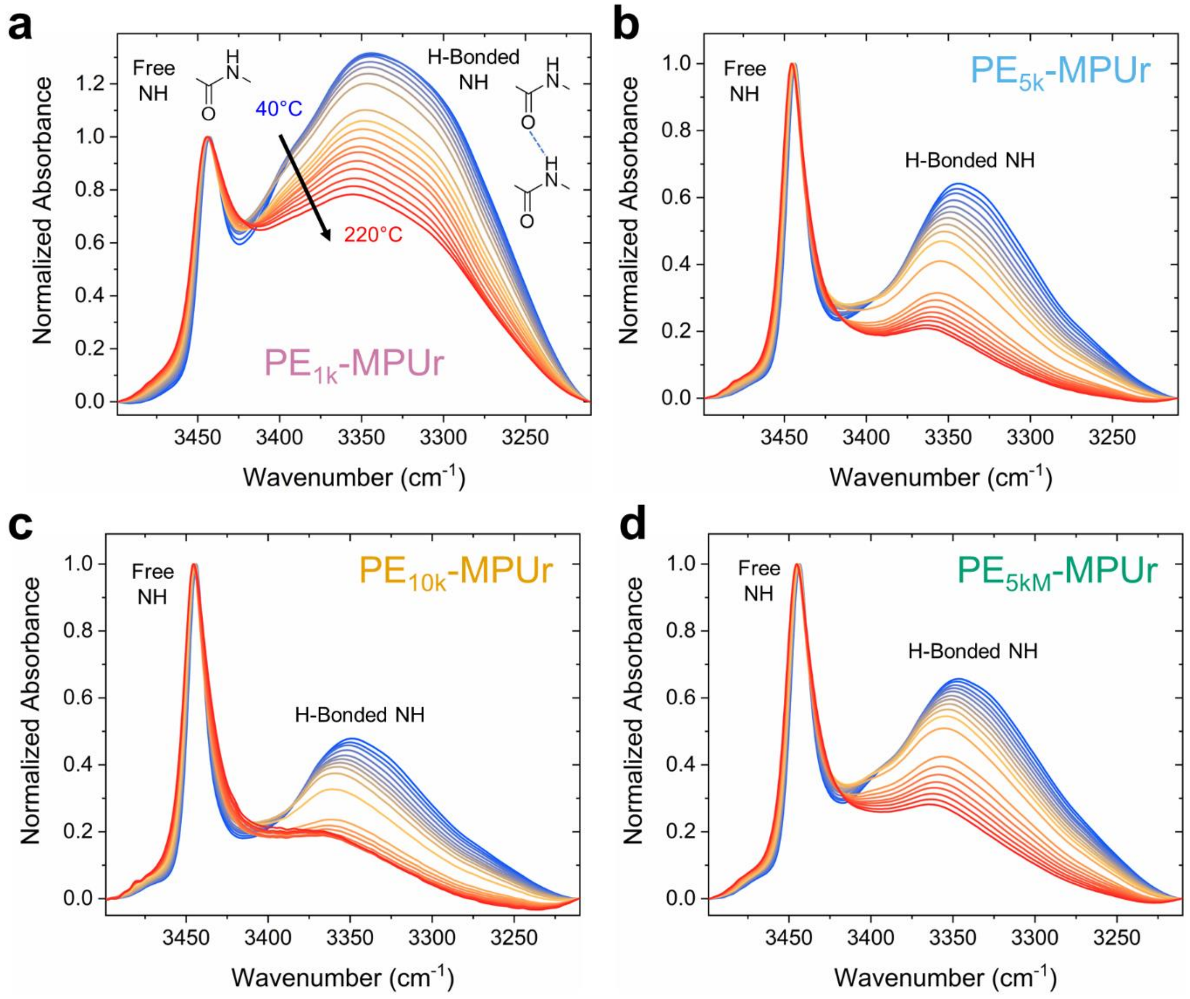

Figure S14. Normalized FTIR absorbance of dynamic HDPE polymers. Normalized FTIR absorbance of the free and hydrogen-bonded NH stretch from 40 °C (blue, top) to 220 °C (red, bottom) in 10 °C steps for **a,** PE_{1k}MPUr, **b,** PE_{5k}MPUr (reproduced from Figure 4a), **c,** PE_{10k}MPUr, and **d,** PE_{5kM}MPUr.

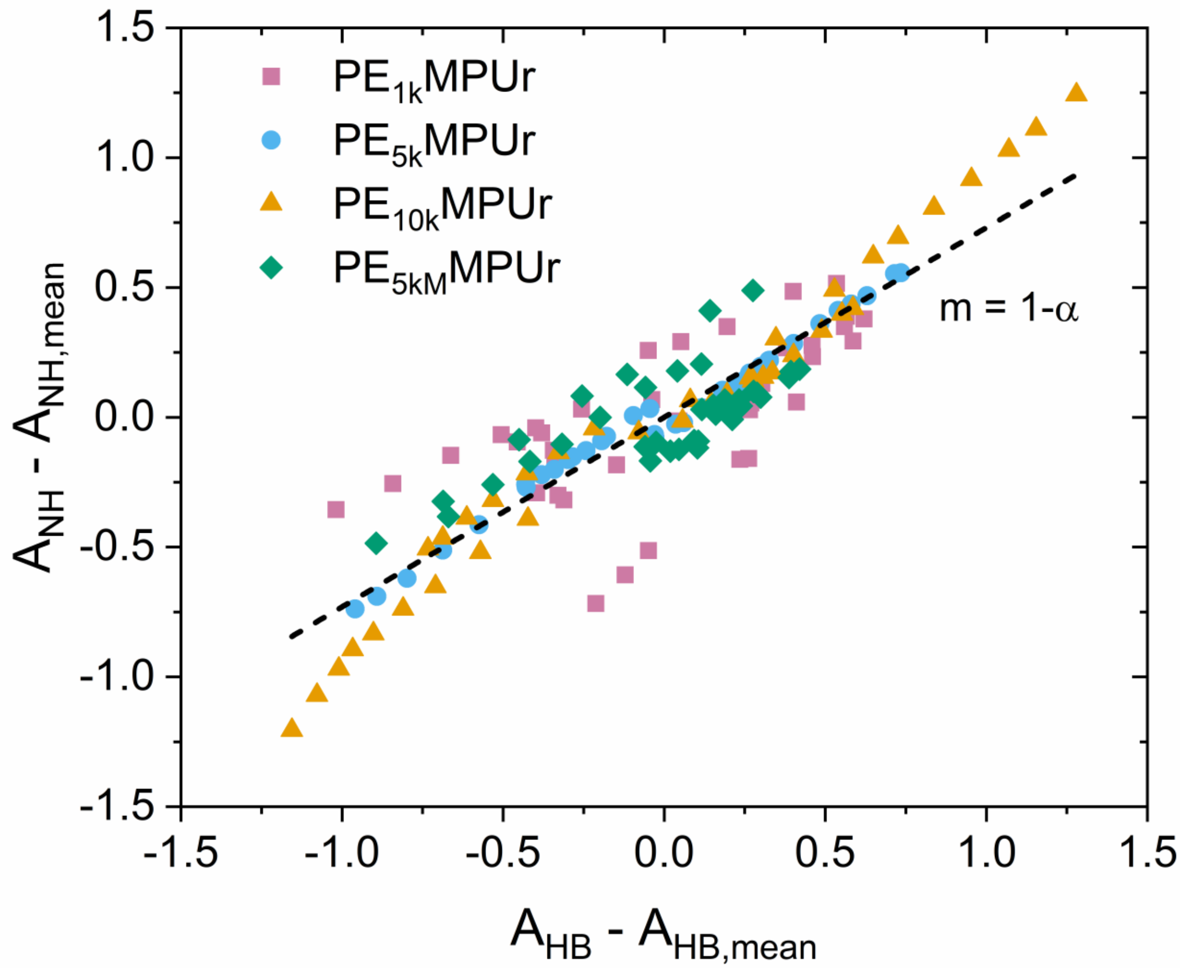

Figure S15. Estimation of molar absorptivity ratio between hydrogen-bonded and free NH. Difference between total area of bonded and free NH stretch and sample mean versus difference between area of hydrogen-bonded NH stretch and mean. Points are colored and shaped based on sample: $PE_{1k}MPUr$ (pink, squares), $PE_{5k}MPUr$ (blue, circles), $PE_{10k}MPUr$ (gold, triangles), and $PE_{5kM}MPUr$ (green, diamonds). The black dashed line shows the extracted slope, which corresponds to $1 - \alpha$, where α is the ratio of molar absorptivity between the free and bonded NH stretch. Linear fit has an R^2 value of 0.82 with a slope of 0.73 ± 0.03.

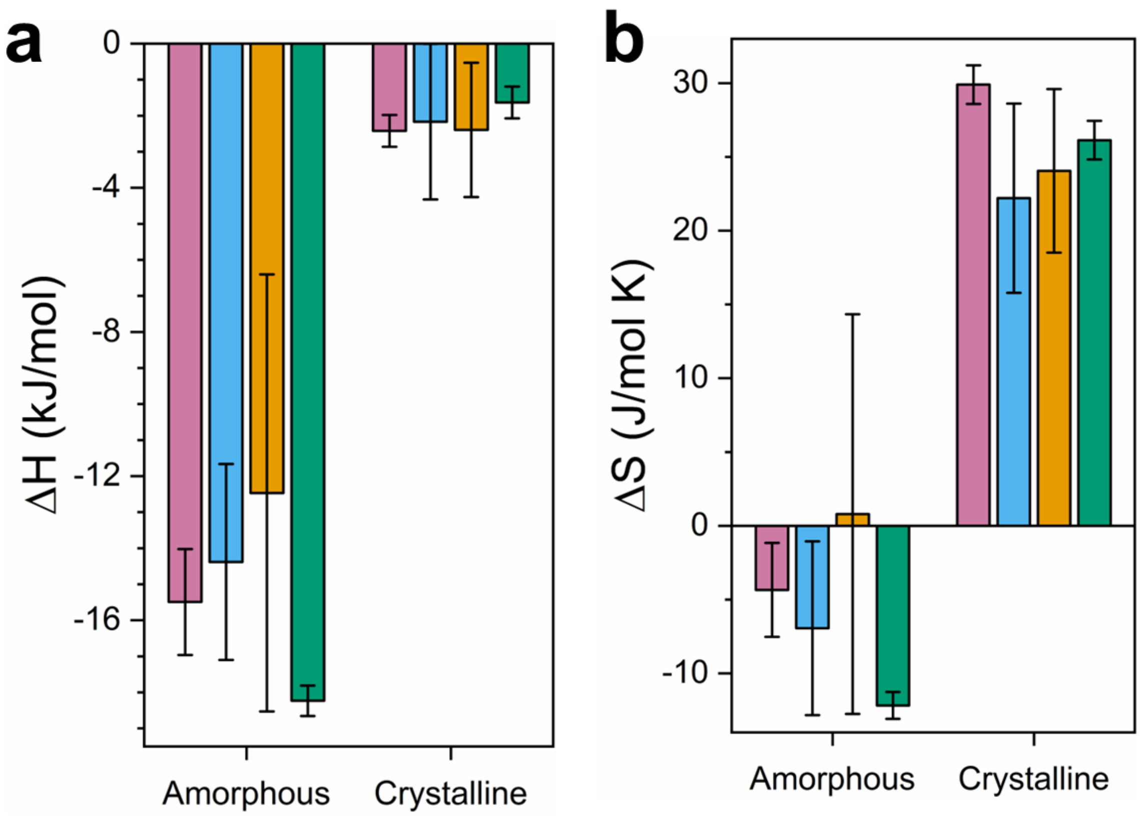

Figure S16. Measured hydrogen bond association parameters. Values for **a,** enthalpy and **b,** entropy in both the semicrystalline and amorphous states for $PE_{1k}MPUr$ (pink), $PE_{5k}MPUr$ (blue), $PE_{10k}MPUr$ (gold), and $PE_{5kM}MPUr$ (green).

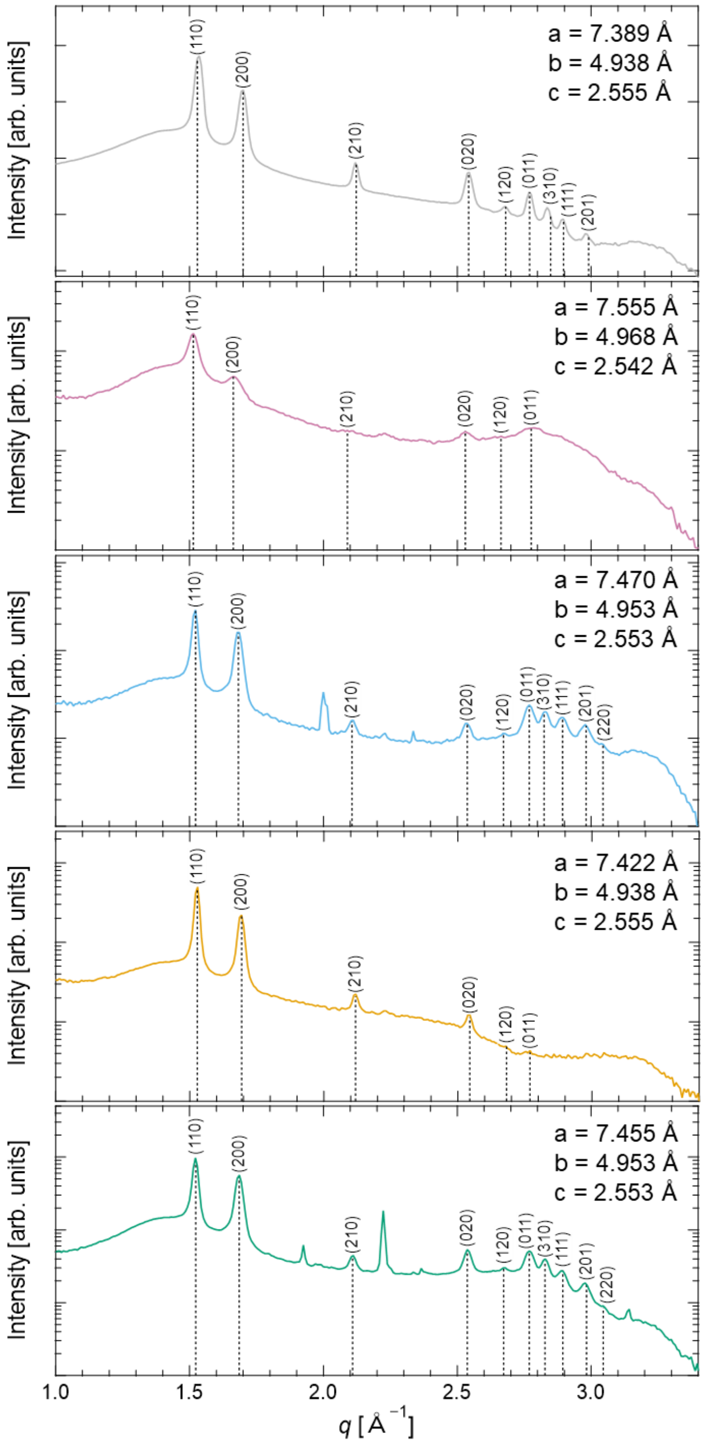

<u>Figure S17. Indexed 1D WAXS data</u>. 1D WAXS for HDPE (light grey, top), $PE_{1k}MPUr$ (pink), $PE_{5k}MPUr$ (blue), $PE_{10k}MPUr$ (gold), and $PE_{5kM}MPUr$ (green, bottom). Dashed lines show peaks indexed to the PE orthorhombic unit cell, with fitted a,b,c unit cell dimensions shown for each polymer. Calculated percent crystallinity values are 71 % (HDPE), 24 % ($PE_{1k}MPUr$), 55 % ($PE_{5k}MPUr$), 61 % ($PE_{10k}MPUr$), and 44 % ($PE_{5kM}MPUr$).

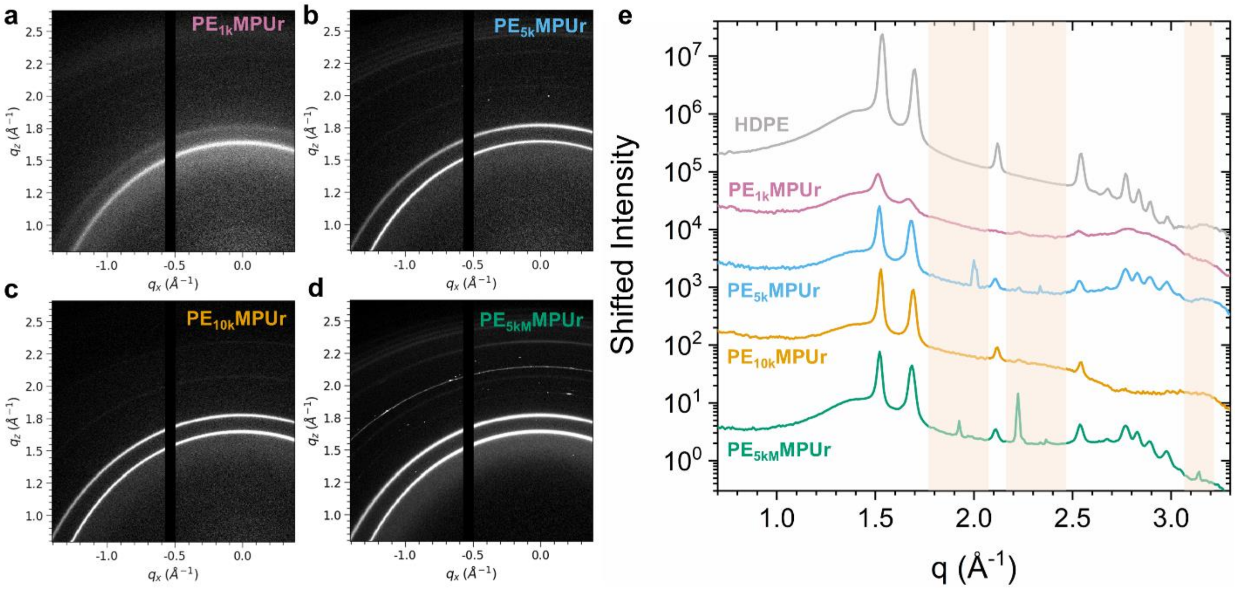

<u>Figure S18. WAXS of dynamic polymers.</u> 2D WAXS images at room temperature for **a,** $PE_{1k}MPUr$, **b,** $PE_{5k}MPUr$, **c,** $PE_{10k}MPUr$, and **d,** $PE_{5kM}MPUr$. **e,** Integrated 1D WAXS data at room temperature for HDPE (grey, top), $PE_{1k}MPUr$ (pink), $PE_{5k}MPUr$ (blue), $PE_{10k}MPUr$ (gold) and $PE_{5kM}MPUr$ (green, bottom). Shaded regions mark areas where additional peaks appear that do not belong to the orthorhombic PE unit cell.

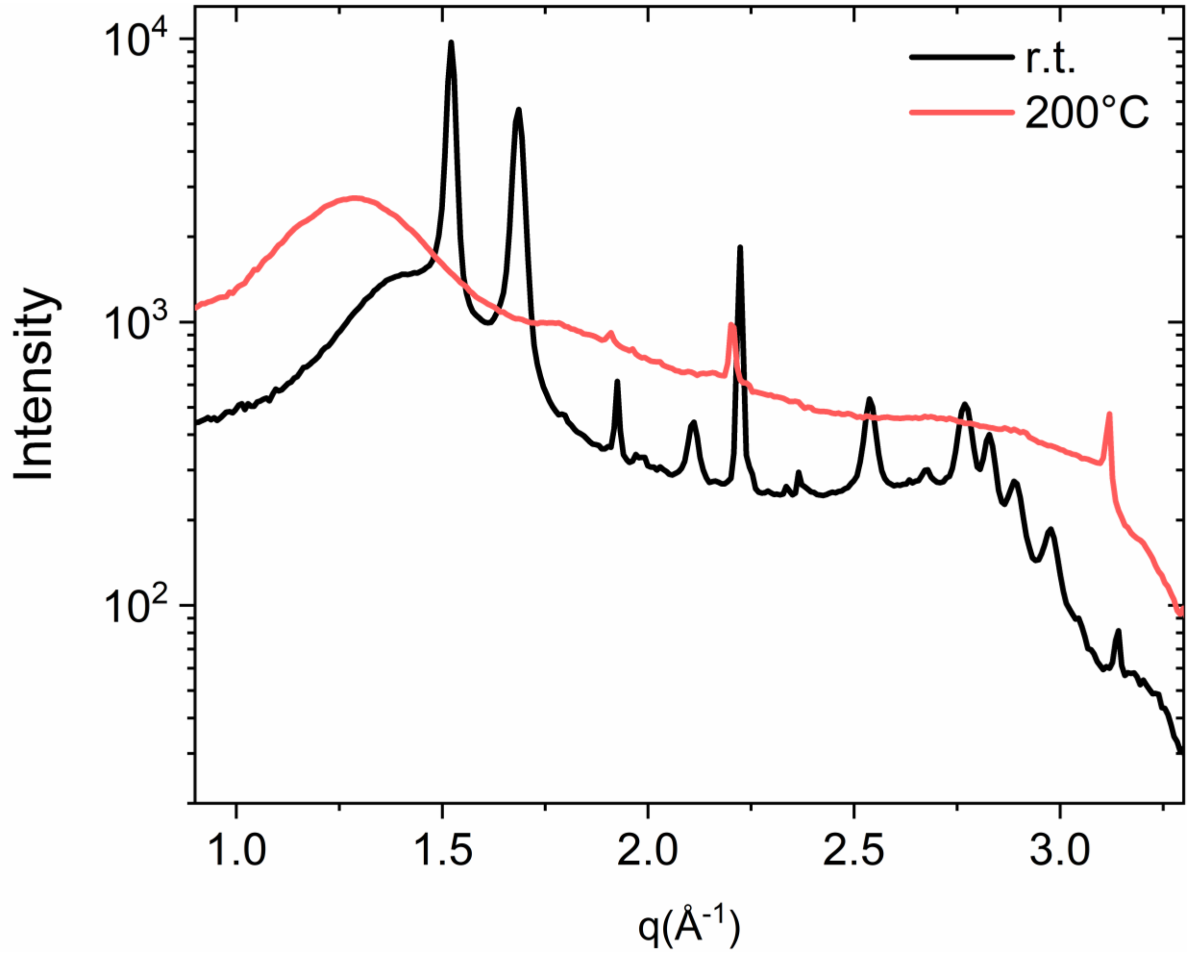

<u>Figure S19. 1D WAXS data for PE$_{5kM}$MPUr.</u> Plotted at room temperature (black) and at 200°C (red), which is above the melting temperature of the PE crystals.

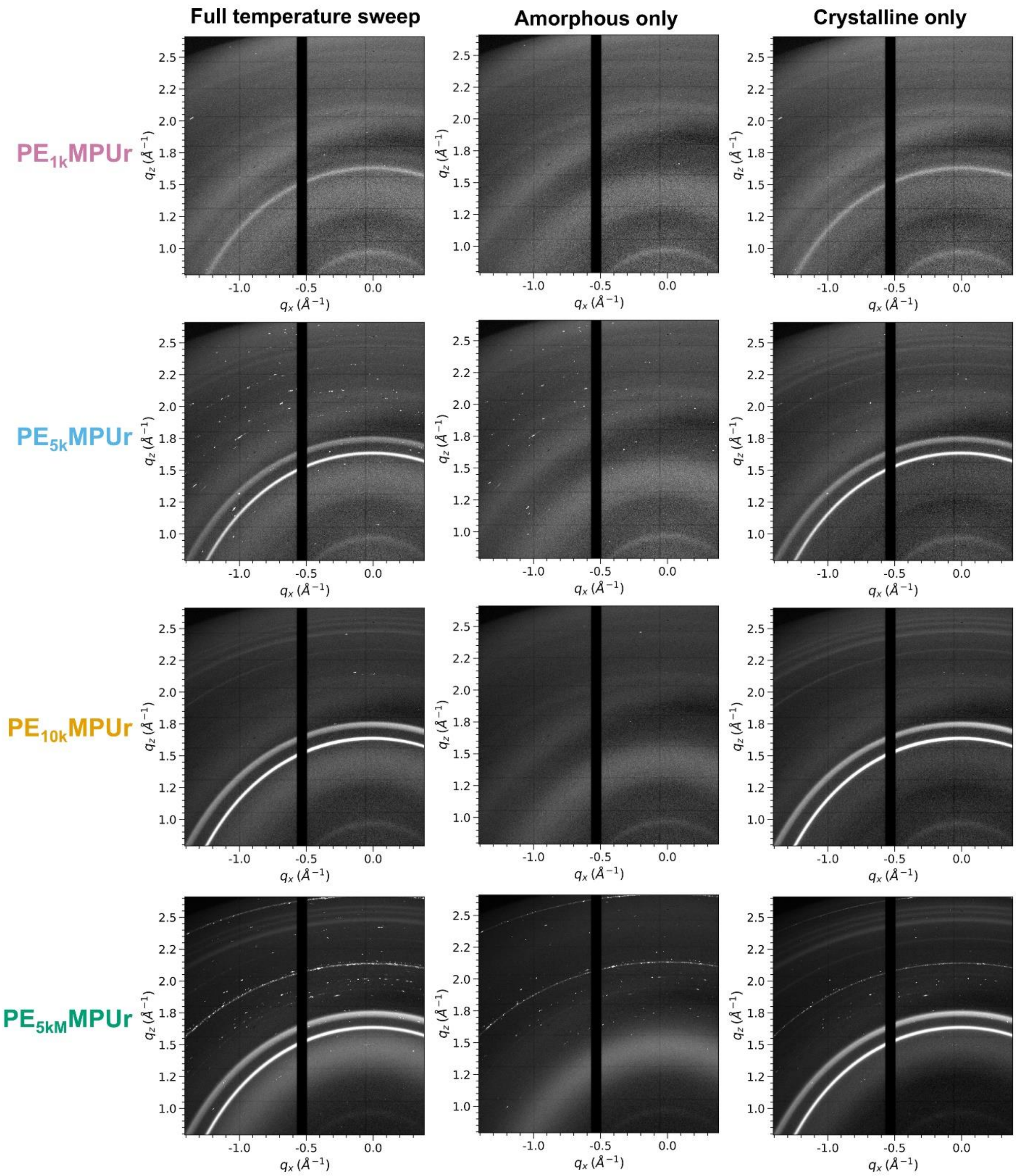

Figure S20. Reconstructed 2D WAXS data. 2D WAXS data that is reconstructed across the entire temperature range (heating and cooling) (left), only when the sample is amorphous (middle), and only when the sample is crystalline (right) for $PE_{1k}MPUr$ (top), $PE_{5k}MPUr$, $PE_{10k}MPUr$, and $PE_{5kM}MPUr$ (bottom). Reconstruction is achieved by keeping the maximum intensity value at each pixel across the entire set of images in each respective group.

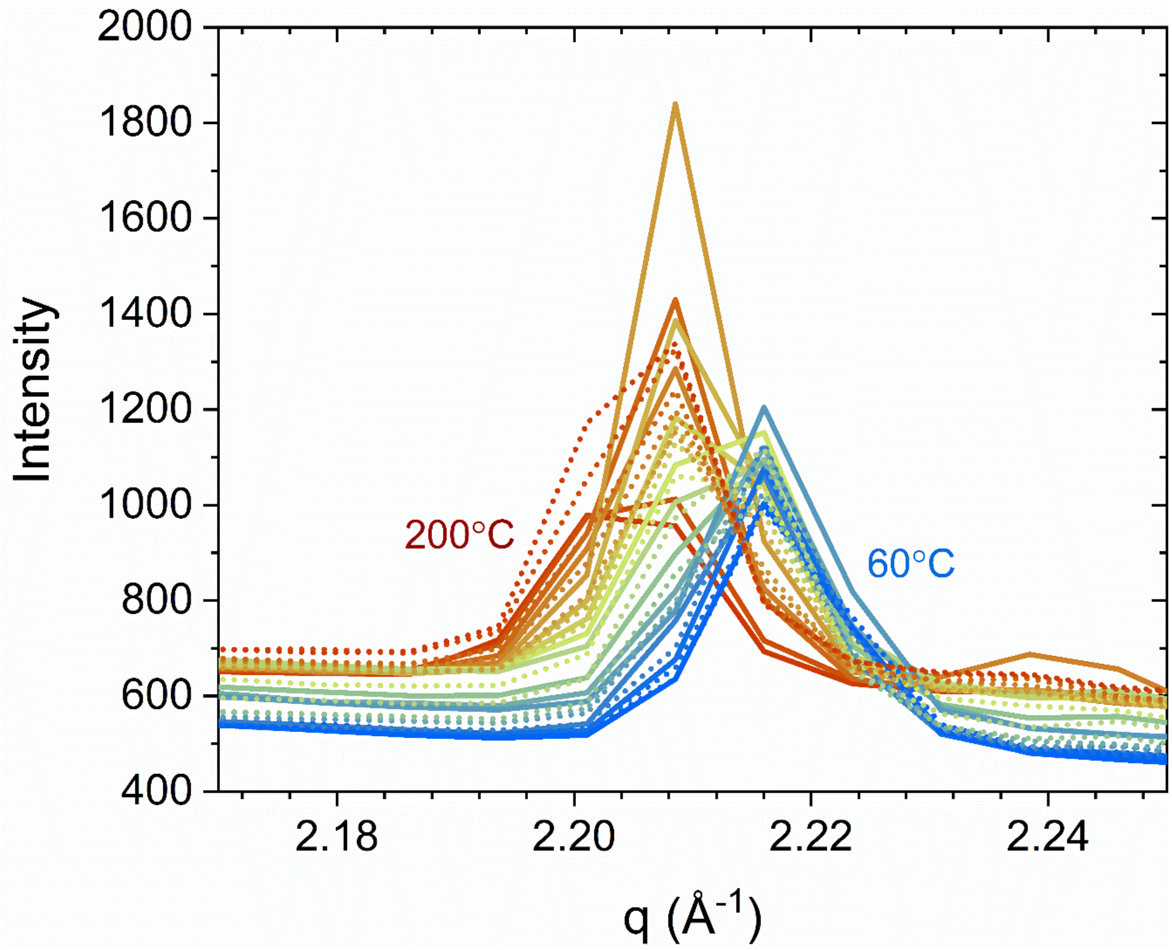

<u>Figure S21. WAXS data for NH…O bond at different temperatures.</u> Plotted for PE_{5kM}MPUr from 60 °C (blue) to 200 °C (red) in 10 °C steps. Melting (heating) traces are solid, crystallizing (cooling) traces are dotted.

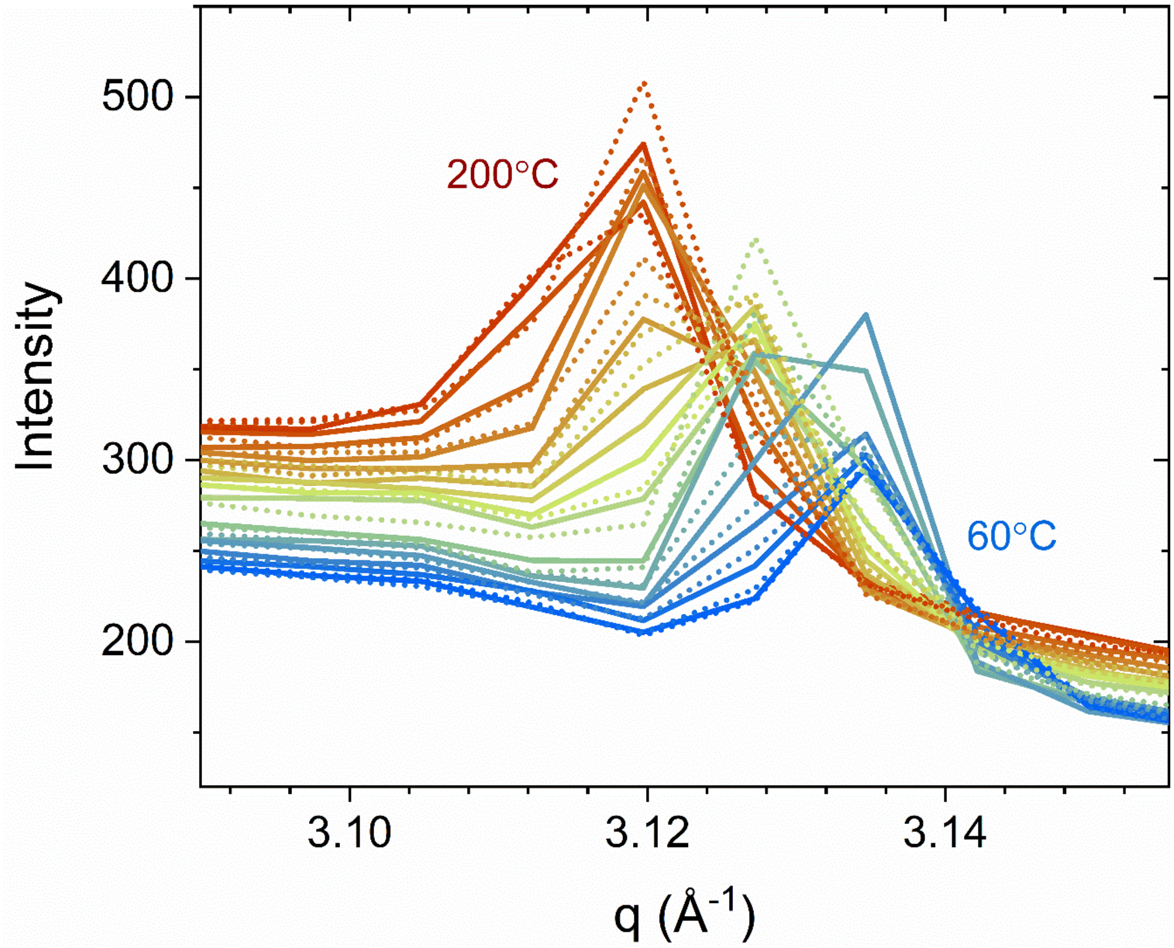

Figure S22. WAXS data for O…H bond at different temperatures. Plotted for PE_{5kM}MPUr from 60 °C (blue) to 200 °C (red) in 10 °C steps. Melting (heating) traces are solid, crystallizing (cooling) traces are dotted.

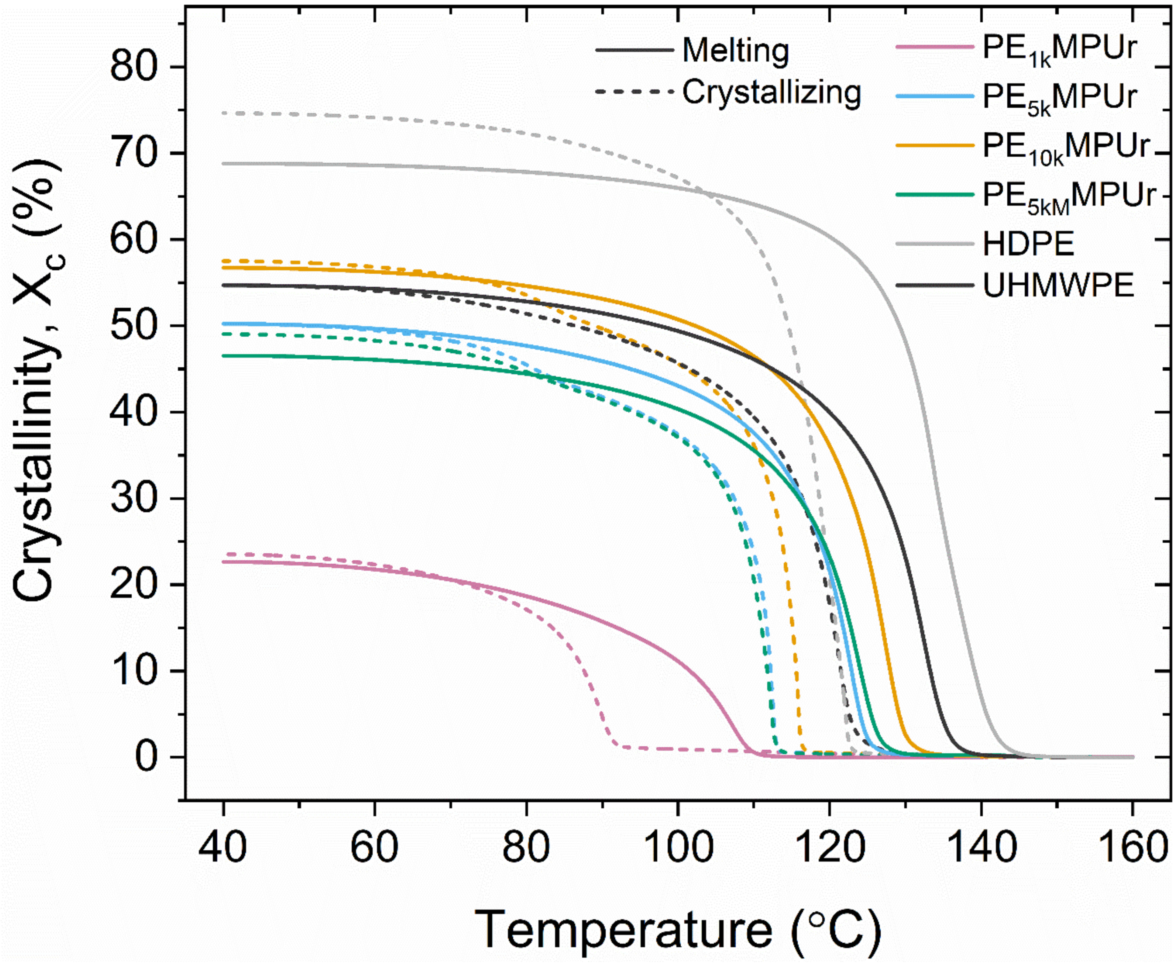

<u>Figure S23. Percent crystallinity versus temperature extracted from DSC measurements.</u> Plotted for $PE_{1k}MPUr$ (pink), $PE_{5k}MPUr$ (blue), $PE_{10k}MPUr$ (gold), $PE_{5kM}MPUr$ (green), HDPE (light grey), and ultra-high molecular weight polyethylene (UHMWPE, dark grey). Melting (heating) traces are solid, crystallizing (cooling) traces are dashed.

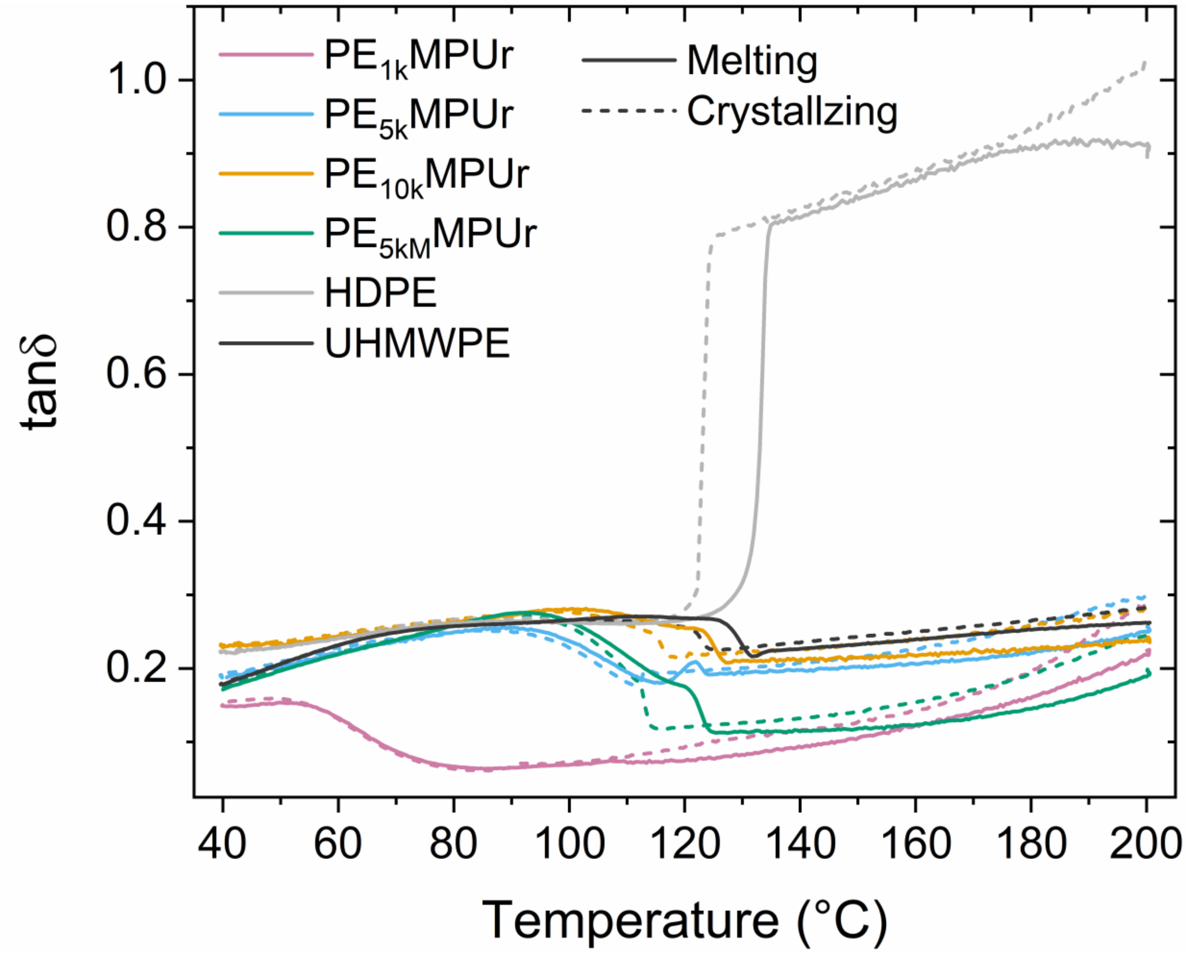

Figure S24. Tan delta versus temperature from rheological measurements. Plotted for $PE_{1k}MPUr$ (pink), $PE_{5k}MPUr$ (blue), $PE_{10k}MPUr$ (gold), $PE_{5kM}MPUr$ (green), HDPE (light grey), and UHMWPE (dark grey). Melting (heating) traces are solid, crystallizing (cooling) traces are dashed.

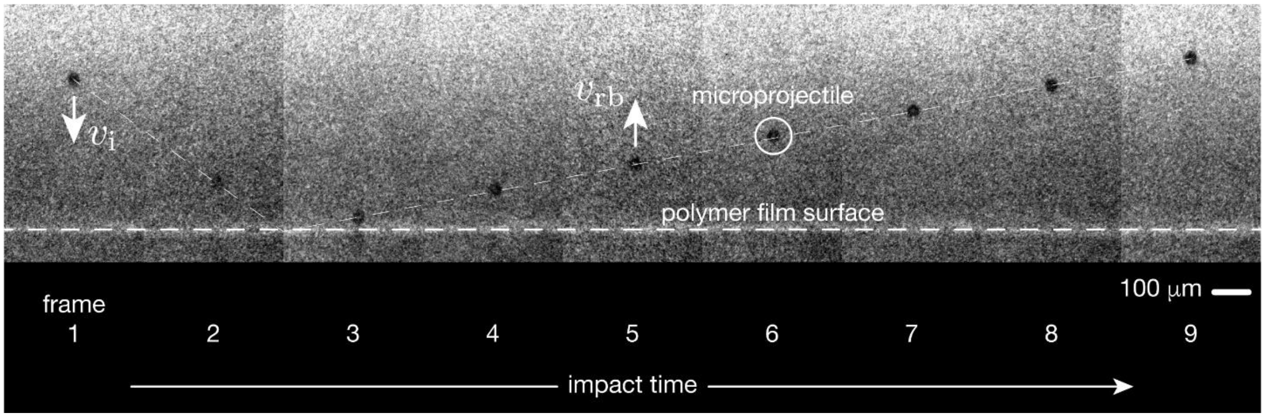

Figure S25. Images of high-strain rate microballistic impact measurements. Timelapse ultrafast camera images of a representative impact event (interframe time is 490 ns). Images capture the trajectory of a 20 μm silica microprojectile as it impacts (v_i = 347 m/s) and rebounds (v_{rb} = 85.7 m/s) off the polymer surface.

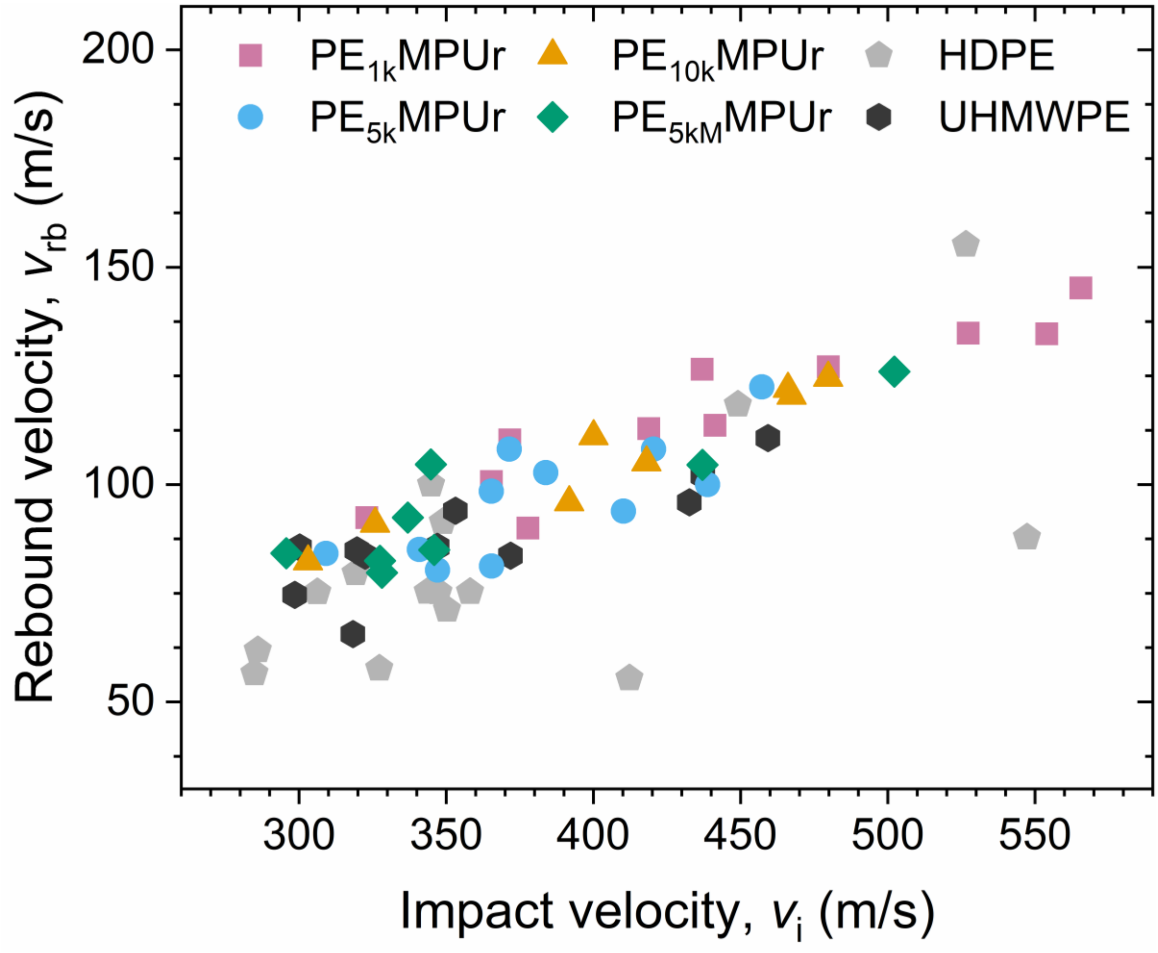

Figure S26. Rebound velocity versus impact velocity. Plot of impact (v_i) and rebound (v_{rb}) microprojectile velocities measured from the ultrafast camera images from which the reduction in kinetic energy is calculated. Data shown from $PE_{1k}MPUr$ (pink), $PE_{5k}MPUr$ (blue), $PE_{10k}MPUr$ (yellow), $PE_{5kM}MPUr$ (green), HDPE (light grey), and UHMWPE (dark grey). Each point represents a unique impact test.

Descriptions of Videos (Video S1-S4)

Video S1. 2D WAXS of PE1kMPUr upon melting. Video is supplementary file named ja5c13586_si_002.avi. Video shows sample WAXS data upon cooling and heating between 200 °C to 60°C at 10 °C/min, where data were collected every 5 s with a 0.5 s exposure time. Temperature labelled on each frame.

Video S2. 2D WAXS of PE10kMPUr upon melting. Video is supplementary file named ja5c13586_si_003.avi. Video shows sample WAXS data upon cooling and heating between 200 °C to 60°C at 10 °C/min, where data were collected every 5 s with a 0.5 s exposure time. Temperature labelled on each frame.

Video S3. 2D WAXS of PE5kMPUr upon melting. Video is supplementary file named ja5c13586_si_004.avi Video shows sample WAXS data upon cooling and heating between 200 °C to 60°C at 10 °C/min, where data were collected every 5 s with a 0.5 s exposure time. Temperature labelled on each frame.

Video S4. 2D WAXS of $PE_5kMMPUr$ upon melting. Video is supplementary file named ja5c13586_si_005.avi. Video shows sample WAXS data upon cooling and heating between 200 °C to 60°C at 10 °C/min, where data were collected every 5 s with a 0.5 s exposure time. Temperature labelled on each frame.
